# A functional investigation of antibody Fc-FcRn variant binding guided by *in silico* free energy perturbation methods

**DOI:** 10.64898/2026.04.28.721095

**Published:** 2026-04-30

**Authors:** Jared M. Sampson, Alina P. Sergeeva, Tianyang Gao, Young Do Kwon, Eswar Reddem, Fabiana A. Bahna, Seetha M. Mannepalli, Baoshan Zhang, Peter D. Kwong, Lawrence Shapiro, Barry Honig, Richard A. Friesner

**Affiliations:** a Department of Biochemistry and Molecular Biophysics, Columbia University, New York, NY, USA, 10027; b Department of Chemistry, Columbia University, New York, NY, USA, 10027; c Life Sciences Software, Schrödinger, Inc., New York, NY, USA, 10036; d Department of Systems Biology, Columbia University, New York, NY, USA, 10032; e Vaccine Research Center, National Institute of Allergy and Infectious Diseases, National Institutes of Health, Bethesda, MD, USA, 20892; f Zuckerman Mind Brain Behavior Institute, Columbia University, New York, NY, USA, 10027; g Aaron Diamond AIDS Research Center, Columbia University, New York, NY, USA, 10032; h Department of Medicine, Columbia University, New York, NY, USA, 10032

**Keywords:** antibody-receptor interactions, binding affinity prediction, free-energy methods

## Abstract

Accurate calculation of energy changes upon mutation is a key requirement for the effective use of computational methods in protein design. In this study, we applied free energy perturbation (FEP) calculations to predict the effects of mutations on the binding free energy between the immunoglobulin subtype G (IgG) antibody fragment-crystallizable (Fc) region and the neonatal Fc receptor (FcRn), an interaction that is primarily responsible for antibody half-life. We assembled an extensive experimental dataset of Fc-FcRn binding affinities for wild-type (*wt*) and mutant complexes, including values from literature and from newly measured results. Starting from a crystal structure of the M252Y/S254T/T256E (“YTE”) Fc variant bound to FcRn, we prepared all-atom models of human IgG1-subtype *wt* and YTE variant Fc-FcRn complexes, adding explicit hydrogens and assigning protonation states for key ionizable residues. Initial results using standard FEP protocols to compute relative binding free energies were promising but exhibited multiple outliers. By accounting for coupling effects for FEP mutations near key histidine residues, we improved the results for several outliers, suggesting such coupling as an important approach for pH-sensitive systems. Further, upon determining new crystal structures of four Fc variants at multiple pH values, we observed subtle conformational changes in unbound Fc; by accounting for these conformational changes in FEP calculations, we additionally improved agreement with experiment. The detailed structural and energetic analyses of the Fc-FcRn system we present here thus provide an accurate energy-calculation framework to enable rational *in silico* design of novel Fc variants.

## Introduction

The neonatal Fc receptor (FcRn) plays a critical role in maintaining the circulating immunoglobulin subtype G antibody (IgG Ab) concentration in humans and other mammals. This homeostatic regulation is accomplished via a recycling mechanism in which, after nonspecific fluid-phase uptake of the antibody by pinocytosis, FcRn binds to the Ab fragment crystallizable (Fc) region in the acidified early endosome [1]. The IgG-FcRn complex is transported back to the cell surface, where, due to reduced binding affinity at serum pH, the complex dissociates, returning the antibody to circulation and freeing the receptor for reuse [1]. This pH-dependent binding to FcRn increases the serum half-life of IgG antibodies to approximately three weeks, from 5-7 days for other antibody subtypes [2]. In addition to controlling IgG antibody concentration, FcRn has a number of other functions, including transport of IgG from maternal to fetal circulation in the placenta, and facilitating IgG absorption from mother’s milk by the infant gastrointestinal epithelium, the function from which its name is derived [1, 3, 4, 5].

Over the past few decades, numerous attempts have sought to engineer the Fc region to improve IgG half-life by increasing binding affinity to FcRn in the endosome [6, 7, 8, 9, 10, 11, 12, 13, 14, 15]. Currently available modified Fc regions have been shown to increase IgG half-life by approximately 2- to 6-fold, but further optimization leading to larger increases in half-life could potentially enhance the utility of antibodies in prophylactic and other long-term applications. While substantial progress has been made in producing Fc variants with altered binding affinities, the current best-performing Fcs have all been identified using experimental display and sorting techniques and may be suboptimal with regard to the binding pH-dependence that would maximize antibody half-life. Ideally, this would involve very high affinity binding to FcRn at acidic pH (~6.0) in the early endosome and complete release back into serum at pH 7.4. The challenge, then, is to design antibodies whose FcRn-binding properties vary significantly over this pH range. However, accounting for protonation state and pH sensitivity presents a substantial obstacle to structure-based rational design efforts, where current computational screening methods are generally empirically fit or trained without consideration of these features.

We have investigated the utility of computational methods, specifically free energy perturbation (FEP) calculations, to design an Fc region that can substantially extend antibody half-life beyond what is obtainable with currently available variants. FEP calculations consist of molecular dynamics (MD) simulations with all-atom explicit solvent and simulate an alchemical transition between two closely related molecular species—in the present case, two amino acid sidechains. We use an implementation of the FEP methodology called FEP+ (Schrödinger, Inc.), which has demonstrated success in the prediction of free energy changes for a range of systems and perturbation types, including residue mutation in protein-protein binding, as well as other applications such as small molecule ligand-receptor binding, protein stability, and protein residue pKas [16, 17, 18, 19, 20, 21, 22].

In the study reported here, we carried out an extensive FEP-based investigation of the effect of protein residue mutation on Fc-FcRn binding. We utilized the high-resolution (2.4 Å) structure of the human Fc-FcRn complex (PDB 6WNA) as the starting point for FEP calculations [23], and supplemented the available structural information from the literature with new crystal structures of unbound apo Fc variants, crystallized over a range of pH values. We employed validated simulation and analysis methods for computing pKa values of key ionizable residues from both the antibody and the receptor, as well as the effects of those pKas on binding affinities [19, 24, 22], utilizing as the energy model the most advanced version of the protein molecular mechanics force field available for FEP+ simulations, OPLS5, which incorporates explicit polarization for a key subset of residues [25].

We evaluated the agreement between theory and experiment by comparing FEP-calculated and experimental relative binding free energies (ΔΔ*G*s) for a large dataset of Fc and FcRn variants, augmenting a curated set of Fc variant cases from literature with a set of newly measured binding affinities, including data for both Fc and FcRn variants. We improved an FEP protocol for calculating the pH-specific effect of mutations on protein-protein binding, as previously proposed [22], via an enhanced treatment of a small subset of mutations which, by virtue of their proximity, are strongly coupled to the protonation states of two key histidine residues that are the putative pH sensors in the system. Simple empirical criteria were used to identify a minimal set of such residues, thus ensuring limited impact on overall computational cost.

When this updated methodology was applied to the entire dataset described above, only a small number of significant outliers (cases where predicted binding ΔΔ*G* differs by more than 2 kcal/mol from experiment) remained. The most likely explanation for these outliers involved mutation-induced conformational changes of either the Fc-FcRn complex, or the isolated Fc. We explored these possibilities by updating our FEP calculations to take the new apo Fc structural information into account. The results obtained here suggest that a systematic effort integrating FEP calculations with structural biology can be used to achieve a robust understanding of the free energy landscape.

## Results

### Preparation of pH-specific all-atom structural models

**Figure 1.**
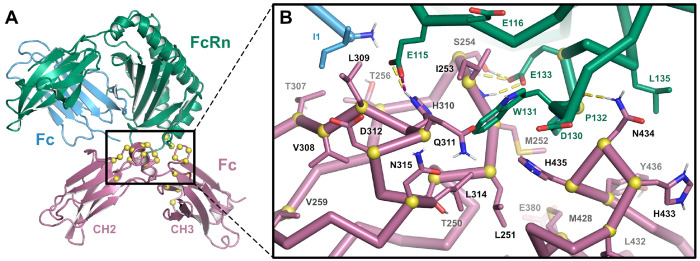
All-atom structural model of the Fc-FcRn complex used for FEP calculations. (A) Ribbon representation of the structural model of the *wt* Fc-FcRn complex prepared from the 6WNA crystal structure, with the FcRn α chain shown in green, β2 microglobulin in light blue, and Fc in violet. Sites investigated using FEP are indicated by yellow spheres. (B) Close up view of the binding interface in the same orientation as indicated by the box in (A). Protein backbone is shown as cylindrical ribbons, key Fc and FcRn/β2M residues are labeled, and *trans* hydrogen bonds and salt bridge interactions between the antibody and receptor are shown as dashed lines.

Using all-atom complex models of wild-type (*wt*) and YTE Fc bound to FcRn prepared as described in Materials and Methods, we calculated pKa values for key ionizable amino acid sidechains at the binding interface using the FEP-based method described by Coskun and coworkers [19]. These calculations were run using either OPLS4 or OPLS5 force fields, with the latter modeling explicit polarization of key groups, including aromatic and carboxylate amino acid sidechains [25]. Table 1 presents computed pKa values for both the *wt* and YTE Fc-FcRn complex models. Based on these pKa values and the experimental pH of 6.0, structural models were updated to include the protonation states of the interfacial ionizable residues indicated in the last column of the table. The final, pH-specific all-atom structural model for the *wt* Fc-FcRn complex is presented in Figure 1.

### Mutational binding affinity dataset curation

We assembled an extensive set of published binding affinities (*K*_D_) for *wt* and variant Fc-FcRn binding interactions at endosomal pH. The variant Fcs included single, double, and triple-mutants relative to either *wt* or M252Y/S254T/T256E (YTE) triple-mutant backgrounds [6, 7, 8, 9, 10, 11, 12, 13, 14, 15]. This dataset included 89 *K*_D_ data points comprising 52 unique variants, and spanned a binding ΔΔ*G* range from −3.59 to +0.77 kcal/mol relative to *wt* Fc-FcRn after converting *K*_D_ values to ΔΔ*G* and averaging across sources. Due to emphasis in the literature on improving binding between Fc and FcRn, most (93%) of these published variant measurements were favorable (with negative ΔΔ*G*, and therefore higher binding affinity) compared to *wt*, with only 5 unfavorable mutation cases.

In an effort to provide additional validation data and broaden our understanding of specific structural regions of the Fc-FcRn binding interface, we performed additional SPR binding affinity measurements for 45 Fc and FcRn variants using anti-HIV-1 monoclonal Ab VRC01 as the *wt* antibody [26]. SPR traces and analyses are included in Supplemental Figure 1, and the new binding data are summarized in Supplemental Table 1. These new measurements included 10 single amino acid substitutions in the YTE Fc background, and 5 FcRn variants binding to *wt* Fc. Altogether the combined dataset contains 134 measurements of 85 unique variants, with ΔΔ*G* values ranging from −3.59 to +2.41 kcal/mol, and are summarized in Table 2. Further analysis of the experimental dataset is shown in Supplemental Figure 2, and the entire combined dataset is included with this manuscript as Supplementary Dataset 1.

### Retrospective FEP results

**Figure 2.**
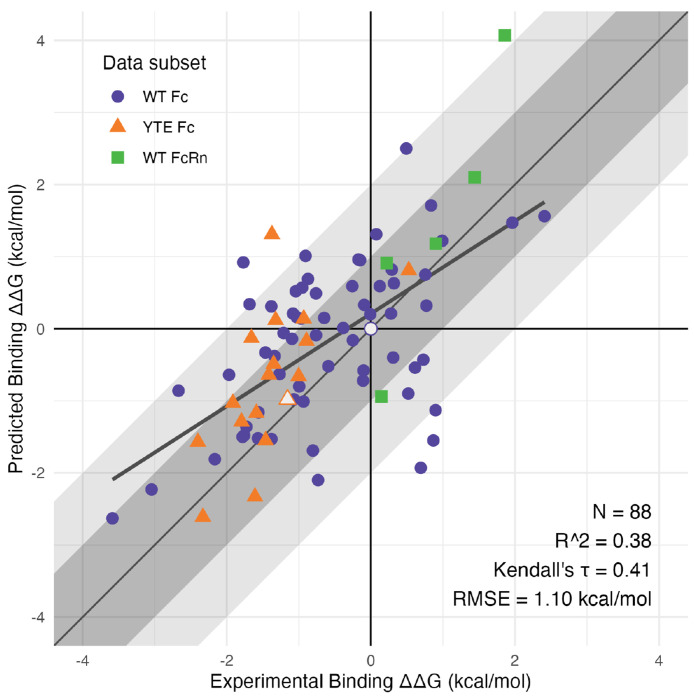
Final retrospective FEP results, showing correlation of FEP-predicted vs. experimental binding ΔΔ*G* values relative to *wt* Fc-FcRn. Point shape and color indicate which model (*wt* or YTE) was used, and which protein (Fc or FcRn) was mutated. White points indicate the results for *wt* and YTE starting models. Overall statistics are shown for the full merged dataset.

We performed 10 ns Protein FEP binding calculations for each Fc or FcRn variant from the experimental dataset with three or fewer mutations relative to either the *wt* or YTE starting models. The variants were divided into three data subsets based on which starting model was used (*wt* or YTE) and which protein (Fc or FcRn) was mutated. The simulations for each variant used the starting model requiring the fewest mutations. For example, the Fc variant M252Y/S254T was accessible as a double mutant from *wt* Fc, and as a single E256T reversion mutant from YTE, so this data point was included in the YTE Fc subset. Final retrospective FEP results for the three data subsets are shown in Figure 2, reflecting binding ΔΔ*G* predictions after the iterative process described in the remainder of the text. In the first stage of this process, ΔΔ*G* results obtained after applying the Protein FEP Groups treatment at pH 6.0 were reported as binding ΔΔ*G* values relative to the *wt* Fc-FcRn starting model, which was also included as a triple reversion mutation variant in the YTE Fc subset. The Protein FEP Groups treatment, originally presented in [22] and described in detail in Supplementary Methods, accounts for the effects of pH in a robust way, effectively weighting the contributions of alternate protonation microstates in the unbound and bound forms. Retrospective FEP results for the full dataset at various stages of our analysis are presented in Supplemental Figure 3, with the above-described preliminary results shown in Supplemental Figure 3A.

### Accounting for the effects of mutations on the ionization states of key histidine residues

With proper weighting of the contributions of protonation microstates for mutated residues accomplished via Protein FEP Groups treatment, we had addressed the most obvious requirements in the computation of the effects of mutation on binding pH dependence. However, in cases where the mutated residue was in close proximity to ionizable residues whose pKa values, and therefore protonation states, changed substantially as a result of the binding process, a more complex treatment was needed.

For variants with mutations near key Fc ionizable residues H310 and H435, we hypothesized that interactions with the mutated sites could alter the His residue pKas, and therefore affect the overall binding affinity of the mutant complex as a function of pH. From a statistical mechanical perspective, treating the sites as coupled required the calculation of binding Δ*G* values for all possible combinations of microstates, as described in the Supplementary Methods. Practically, this corresponded to sequential multiple mutation calculations such that the FEP perturbation map—a network graph in which unique protein states are represented as nodes and FEP perturbations between them as edges—included all possible microstates of the mutated residue as well as the two His pH sensors, where the total number of microstates for a given variant was the product of the individual numbers of microstates possible for each coupled titratable site.

We investigated seven cases this way, namely Fc mutations L251N, M252R, M252H, I253M, H435K, H435Y, and L309D/Q311H/N434S (“DHS”). These cases were selected as described in Materials and Methods based on empirical criteria for solvent accessibility and distance from the ionizable nitrogen atoms in the H310/H435 sidechain imidazole groups. The different identities of the mutated residues at these coupled sites led to varying levels of complexity in the setup of the FEP calculations. For example, in the context of L251N, I253M, or M252R, 9 microstates were required, enumerating all combinations of H310 and H435 protonation states (HID/HIE/HIP), which were included in both the *wt* and mutant contexts. Each distinct combined microstate was represented as a node in the FEP perturbation map. The M252H mutation introduced a third His residue, therefore requiring 27 nodes in the fully coupled FEP map corresponding to that variant. The H435K and H435Y mutations each eliminated one of the coupled His sites in question (H435), thus reducing the number of microstates in the mutant context, though in the case of H435K this was replaced with a titratable lysine sidechain. The most complex and computationally expensive variant treated in this manner was DHS, with 54 possible microstates for the L309D/Q311H/N434S triple mutant alone, with additional nodes representing microstates for the intermediate single and double mutants. Accordingly, to reduce computational costs, an intermediate L309D/Q311H starting model was used for the DHS simulations. This reduced the number of perturbations required to sample all the microstates, but relied on the assumption that the neutral N434S mutation, with a Cβ-Cβ distance of about 6 Å to the closest of the coupled titratable sites (H435), did not substantially affect the protonation states of any of these titratable residues. The FEP perturbation map prepared for the DHS variant alone initially contained over 350 perturbation edges. However, the edges in the automatically generated map were highly redundant, allowing the systematic removal of approximately half of the edges before submitting the calculation, leaving 109 nodes and 171 edges, which was sufficient to fully connect the graph, with each node included in a closed cycle containing 4 or fewer nodes. An illustrative example of these FEP graphs is presented in Supplemental Figure 4.

After performing the additional calculations to account for the coupling of these seven variants to H310 and H435, we observed a range of effects. While the effect for the L251N was negligible and the prediction for M252H resulted in a higher absolute error by 0.5 kcal/mol, M252R and I253M showed modest improvements relative to uncoupled calculations, with absolute errors decreasing by about 0.2 and 0.6 kcal/mol, respectively. The H435K and H435Y variants also demonstrated some improvement when the effects of coupling to H310 were included, with absolute errors reduced by 0.7 and 0.1 kcal/mol, respectively. The DHS variant exhibited the largest coupling effect, with a reduction in absolute error of about 1.8 kcal/mol. A summary of the changes in predicted binding ΔΔ*G* for these variants can be found in Supplemental Table 2, and the direction and magnitude of the ΔΔ*G* shifts are shown as vertical tails in the right panel of Supplemental Figure 3B.

When binding ΔΔ*G* values from these His-coupled cases were merged into the full dataset and compared to experiment, the resulting *R*^2^ of 0.34 and RMSE of 1.15 kcal/mol were comparable to previously reported protein binding FEP results [17, 18, 22]. However, there remained a small number of significant outliers, including a cluster of cases with mutations on the Fc 252-256 loop exhibiting similar “overbound” behavior—with predicted ΔΔ*G* more favorable than the experimental value, as seen at the lower right of Supplemental Figure 3B—which we decided to investigate via crystallography and further FEP calculations.

### Conformational heterogeneity

For many residues in an FEP simulation, the correct, lowest-energy region of phase space is likely to be already present in, or readily accessible from, the starting conformations of the *wt* and mutant proteins. However, for a small (but potentially significant) subset of residues, the lowest free energy structure in the context of either the complex, or the isolated protein in solution, may not be properly sampled in the course of a typical-duration FEP simulation. Ideally, in the absence of variant-specific experimental structures, one would use protein structure prediction and refinement methodology to identify the most likely starting conformation for every residue. In practice, an approach of this type is not yet feasibly within the capabilities of current refinement algorithms. Consequently, we adopted an iterative methodology in which we initially relied on the FEP simulations to sample the mutant conformational phase space, identified outliers in binding affinity prediction compared to experiment, and investigated the outliers via both computational and experimental means.

### Conformational flexibility in Fc monomer structures

We hypothesized that the observed errors for the overbound subset of outlier cases arose from inadequate representation of the unbound Fc protein due to the existence of a lower-energy conformation that was not sampled in FEP simulations. To test this hypothesis, we determined crystal structures of *wt* Fc and three Fc variants associated with outlier cases (M252H, M252R, I253M) in their unbound apo forms, each at multiple pH values (see Materials and Methods for details on crystallization conditions). Crystallographic data collection and refinement statistics for the 9 structures are presented in Supplemental Table 3.

**Figure 3.**
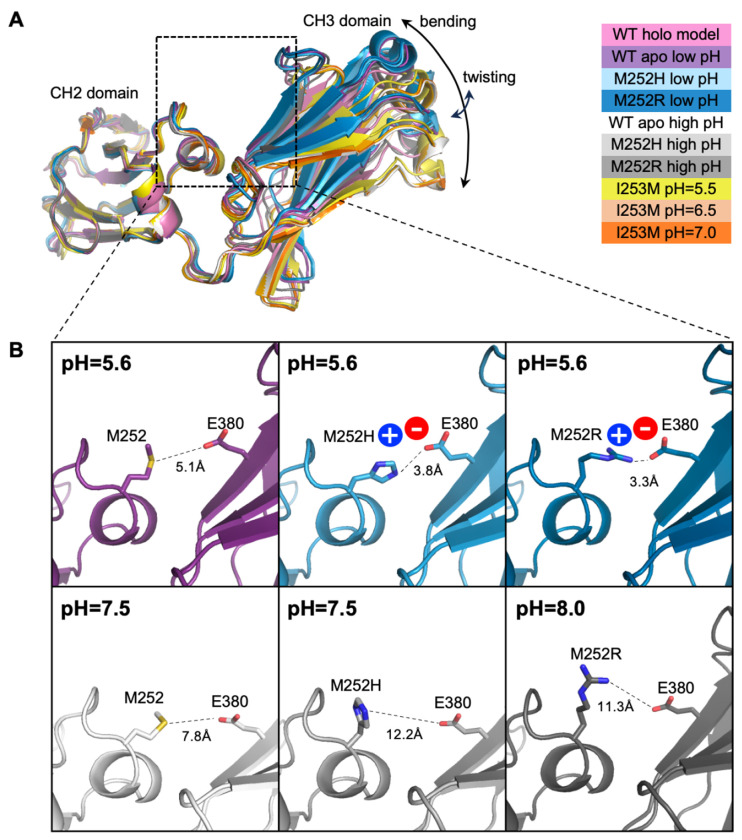
Conformational changes in the Fc protein induced by mutation or pH variation. (A) Ribbon representation of Fc monomer structures crystallized in this study, superimposed on the CH2 domain of the *wt* Fc in the bound state (holo, pink). A detailed structural comparison, including the quantified angle and displacement of the CH3 domain, is provided in Supplemental Table 4. (B) Close-up views of the Fc region highlighted by the dashed box in (A). Residues contributing to enhanced electrostatic interactions in the mutated environment are shown as sticks. Distances between the closest heavy atoms of Fc residues 252 and 380 are shown as dashed lines, with salt bridge formation indicated by circles labeled with residue charges. Structures are color-coded according to the legend in (A). At high pH (bottom row), the distance between residues 252 and 380 is greater compared to low pH (top row).

All apo Fc crystal structures were solved as Fc homodimers; however, monomer representations are shown in Figure 3A to illustrate the overall conformational changes affecting CH2-CH3 interdomain orientation. With CH2 domains aligned, the CH3 domains exhibited translational displacements varying from 0.1 to 6.0 Å, and angular shifts ranging from 0.5° to 20.7° relative to three different reference structures (Supplemental Table 4).

**Figure 4.**
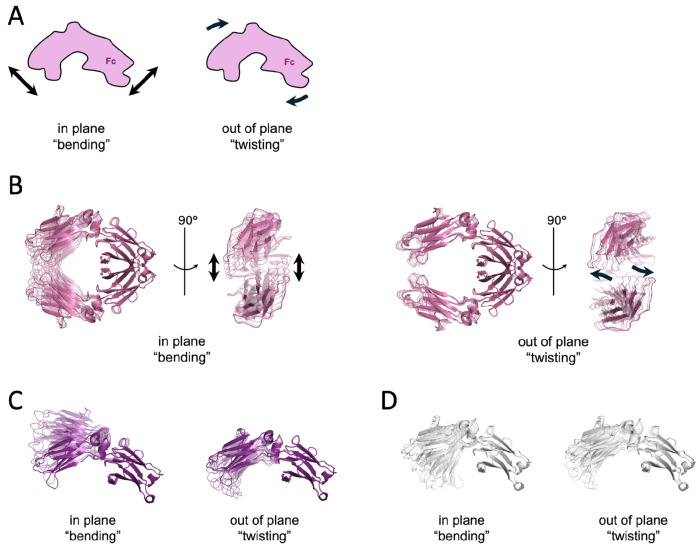
Normal mode analysis of the Fc antibody region. (A) Schematic representation of the lowest frequency mode (“bending”) and the second lowest frequency mode (“twisting”) of Fc. (B-D) Ribbon representations of normal mode motions in the Fc dimer structure (B, pink, PDBID: 4N0U), and two *wt* Fc monomers at low pH (C, purple, PDBID: 9CXL) and high pH (D, white, PDBID: 9CRT) calculated using NOLB (see Materials and Methods) and visualized via PyMOL. Structures are superimposed on the CH3 domain for easier visual comparison.

Normal mode analysis was performed on Fc chains from the original holo model, each new unbound Fc structure, and a dimer Fc model prepared from a lower resolution YTE Fc-FcRn structure (PDB 4N0U). In all structures (both monomers and the dimer), the lowest-frequency mode corresponded to a hinge-like “bending” motion, analogous to the symmetric in-plane bending vibrational mode of a water molecule. The second-lowest-frequency mode represented an out-of-plane “twisting” motion. Linear combinations of these two lowest-frequency modes could describe transitions between all observed structures. The bending mode predominantly contributed to the transition between holo and apo *wt* structures, while the twisting mode appeared to play a role in the transition between low and high pH apo *wt* structures. Regardless of the oligomeric state (monomeric or dimeric Fc), the two lowest-frequency modes remained consistent across both *wt* and mutant proteins and accounted for the structural variability observed in crystallographic data. The observed conformations in the new apo Fc structures corresponding to these domain-level “bending” and “twisting” motions of the Fc molecules are depicted in Figure 4.

The three variant apo Fcs (M252H, M252R, and I253M) contained mutations at adjacent positions on the same structural loop of the CH2 domain. While the loop backbones in both low- and high-pH structures adopted conformations similar to the holo complex structure, we observed differences in sidechain orientations of the mutated residues attributable to shifts in relative domain orientation (Figure 3B). In the low-pH M252H and M252R Fc structures, the mutant His and Arg sidechains each interacted with negatively charged Fc residue E380 from the neighboring CH3 domain, forming a salt bridge and bringing the CH3 domain closer to the CH2 domain in these apo Fc structures compared to the holo conformation. Notably, this low-pH apo Fc conformation introduced clashes when superimposed onto the holo complex, between the Fc CH3 loop containing residue N434 and the H435 pH sensor, and the FcRn loop containing D130. However, these clashes were able to be resolved via loop relaxation (RMSD <0.2Å), and therefore would likely not preclude binding. Based on these observations, we concluded that at low pH, the salt bridge interaction between E380 and a positively charged Arg or His residue in place of a hydrophobic Met at Fc position 252 engenders a change in the apo Fc shape which must then adopt the holo conformation in order to bind FcRn efficiently.

**Figure 5.**
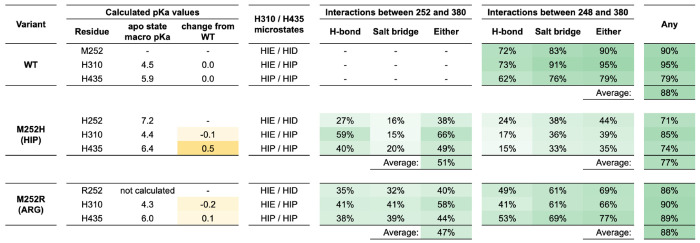
Calculated pKas and persistence of key interactions at the apo Fc CH2-CH3 domain interface in MD simulations of *wt*, M252H, and M252R variant apo Fc. FEP-calculated macro pKa values for His residues at positions 252, 310, and 435 are reported and used to determine 3 relevant microstates to simulate by MD. The fraction of MD frames exhibiting the indicated interactions between either positively charge HIP/ARG252 or LYS248 and GLU380 are shown for simulations set up with the indicated fixed protonation states.

To assess how these interactions behaved in solution, we performed 100 ns MD simulations for the M252H and M252R variants and quantified hydrogen bonds and salt-bridge formation involving E380, as presented in Figure 5. Both variants maintained direct contacts between residue 252 and E380 in roughly half of the trajectory frames. In addition, each variant showed frequent E380 interactions with K248, indicating that the negative charge of E380 is often stabilized by either residue 252, residue 248, or both simultaneously. These MD results reinforce the crystallographic findings by showing that protonated H252 or R252 engages E380 repeatedly in solution, stabilizing local conformations that alter the CH2-CH3 orientation and differ from those sampled by the *wt* Fc.

**Figure 6.**
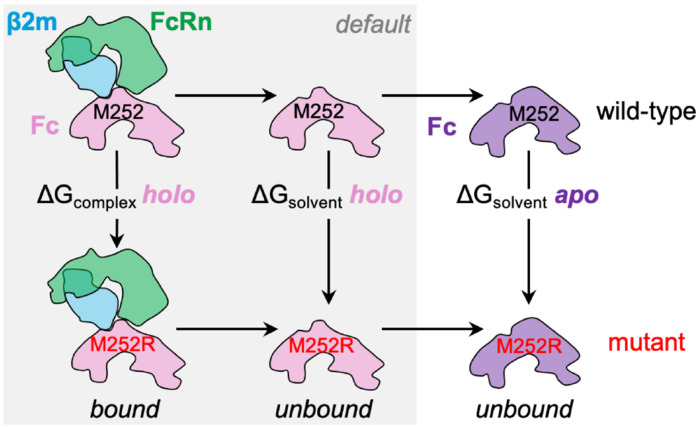
Thermodynamic cycle for FEP calculations of the M252R mutation in Fc protein and its effect on FcRn binding. Alchemical transformations for each mutation are simulated in two environments: the bound state, where Fc is in complex with the FcRn/β2m heterodimer; and the unbound state, where Fc is modeled alone. The default FEP protocol assumes that global Fc conformation is similar in both states. To address significant conformational changes observed in apo Fc crystal structures, we employed an extended thermodynamic cycle that explicitly accounts for structural differences.

The M252R, M252H, and I253M mutations in Fc weaken binding to FcRn, as indicated by a loss of 0.7-0.9 kcal/mol in binding free energy in SPR measurements. However, our initial standard-protocol FEP simulations, employing a thermodynamic cycle that assumed Fc to exist in the same conformational basin when bound to FcRn and in isolation, predicted these mutations to be strongly stabilizing. To account for the conformational variability seen in the crystal structures, we substituted solvent Δ*G* values calculated using an apo Fc model in place of the original, holo-conformation solvent Δ*G*. This change is illustrated in Figure 6, which compares the standard thermodynamic cycle (shaded region in the figure) with the modified version accounting for the conformational change, using the M252R mutation as an example. For mutations far from the CH2-CH3 interface, we expected the effect of this substitution to be minimal, at the noise level, but for key cases like M252R and M252H, we hypothesized that the effect could be substantial.

That there was no clear relationship between pH and the conformational variation in the apo Fc structure (Supplemental Table 4), and the transitions among these structures aligned with the lowest-frequency, and therefore most easily accessible, normal modes (Figure 4) suggested that all of the observed apo and holo Fc conformations were likely to be sampled at room temperature, with comparable energies and similar population weights in the conformational ensemble. Therefore, we made the assumption that the transition between the apo and holo conformations for the *wt* protein incurs no energetic penalty. However, we noted the M252R and M252H mutations had the potential to shift the equilibrium toward the apo conformations, and it is these energy differences that we wished to uncover by using an apo-conformation starting model for Fc solvent leg simulations.

The low-pH apo Fc structures for the *wt*, M252H, and M252R variants were highly similar, with Cα RMSD below 0.4 Å. Therefore, we selected the highest-resolution of these crystal structures (2.2 Å, M252R low pH) to construct all-atom apo-conformation *wt* and YTE Fc models. We performed solvent leg-only FEP simulations for the full mutational dataset using these new apo Fc models and recalculated binding ΔΔ*G*s as the difference between the original (holo) complex leg Δ*G* and apo Fc solvent leg Δ*G*. For the M252H variant, in the dominant HIP protonation state (as determined using FEP-calculated macro pKa values of 6.3 and 8.3 in unbound and bound states, respectively, produced by the Protein FEP Groups output), the apo Fc solvent leg was ~0.7 kcal/mol lower in energy than the corresponding holo Fc solvent leg, resulting in a less overbound ΔΔ*G* relative to experiment. The M252R variant exhibited a larger shift of ~1.0 kcal/mol. With Groups treatment, the weighted average over the three possible His protonation states for M252H (HIP, HIE, HID) reduced the magnitude of the improvement compared to the single-state estimate, but overall, the use of the apo Fc model in the solvent legs reduced errors relative to experiment. After including the apo Fc solvent legs to account for conformational changes in Fc between bound and unbound contexts for all mutations, we observed that most cases were not affected by the change, many were improved to varying degrees, and relatively few cases produced somewhat larger errors, as indicated by the vertical tails shown in Supplemental Figure 3C.

Crystal structures of I253M variant Fc were solved at three different pH conditions (5.5, 6.5, 7.0), all of which were highly similar (RMSD <0.6Å), indicating no significant pH-dependent conformational changes. These structures also more closely resembled the *wt* structure at high pH rather than the *wt* at low pH (Supplemental Table 4). To further investigate the impact of apo conformational differences in computational simulations, we tested two different *wt* conformations in corrected solvent leg simulations: 1) the low-pH *wt* structure (as described above); and 2) the low-pH I253M structure. In each case, calculated solvent leg Δ*G* values remained very similar to the holo-conformation value (approximately −13.5 kcal/mol), which was expected given the largely unchanged local environment near the mutation across conformations. Because using the I253M-specific apo Fc model for the solvent leg did not improve the FEP prediction for this outlier case, in our final FEP results the reported ΔΔ*G* for the I253M Fc variant used the same *wt* apo Fc model as the rest of the dataset.

In the preceding paragraphs, we demonstrated improvement in predicted ΔΔ*G* values by accounting for global conformational differences in the unbound apo Fc structure relative to the holo complex. A second type of error due to incomplete sampling can arise if the FEP simulation is unable to access the lowest free energy mutant sidechain conformation. An issue of this type occurred for the T307W variant when using the low-pH apo crystal structure of the M252R mutation as a starting point, due to the presence of different rotamer conformations of the mutant Trp residue between apo and holo structures. This problem was readily remedied by modeling a different initial rotamer state for the sidechain, as discussed in Supplementary Results and accompanying Supplemental Figure 5, Supplemental Table 5, Supplemental Table 6, and Supplemental Table 7, and presented with the ΔΔ*G* correction shown as a vertical tail in Supplemental Figure 3D. We note that sampling errors of this sort could be avoided by incorporating state of the art conformational sampling algorithms when generating models for the mutant residue to locate the lowest mutant free energy structure, but as this procedure is rarely needed, these additional calculations are not part of the standard FEP workflow.

After incorporating this best-estimate binding ΔΔ*G* for the T307W variant, the results comprised the final retrospective FEP dataset presented in Figure 2 for all 88 experimental mutations. The *R*^2^ value of 0.38 and RMSE of 1.10 kcal/mol were comparable to the results obtained from previous protein binding FEP studies [17, 18, 27, 22]. Although a few predictions remained with absolute errors in the range of 2 kcal/mol, this could be expected in the distribution of FEP deviations from experiment for this size of dataset. The largest remaining outlier was I253M, for which we do not have an explanation—though for which we did remeasure the experimental *K*_D_, confirming the original value using different equipment and reagents (data not shown). Lastly, we note that the final outlier rate for the full Fc-FcRn dataset was less than 2%, which would not represent a barrier to practical antibody engineering applications.

## Discussion

Here we have presented a study of the Fc-FcRn system investigating the effects of amino acid mutations on binding affinity. We approached the system both experimentally through SPR affinity measurements and X-ray crystallography, and computationally via Protein FEP calculations and additional structural and trajectory analyses. The experimental binding affinity measurements presented here provide a substantial addition to the diversity of low-pH mutational binding data in the literature; in particular, the inclusion of several destabilizing variants extends the range of ΔΔ*G* values, allowing a more meaningful assessment of the correlation between predicted and experimental results. We expect the full mutational low-pH binding ΔΔ*G* dataset provided in the supplement to be useful in future studies of this important

By looking at specific cases that were improved at each stage of our calculations, we were able to identify important concepts to the function of the system. For example, the FEP-calculated pKa observed for Fc H435 in the bound context was higher using the OPLS5 force field compared to OPLS4, preferring HIP at pH 6.0. This is in better agreement with the consensus understanding of the mechanism for Fc-FcRn pH dependence, which depends on protonation of key Fc His residues H310 and H435 in the complex, and suggests that pi-pi or cation-pi interactions with nearby FcRn residue Trp131 may be critical to stabilizing the charged H435 sidechain. This is an important finding, and will undoubtedly be useful for other similar cases going forward.

Additionally, although not required for most cases, for certain mutations near or involving the key His residues, we found it was important to consider the effect of the mutations on the distribution of protonation microstates among these coupled titratable sites. In an extreme case, the half-life-extending DHS variant [14], which not only contains mutations near these two key histidines, but introduces two additional ionizable amino acid sidechains (D309 and H311) at the loop positions immediately before and after H310 in the primary amino acid sequence, was able to be successfully modeled—albeit at great computational expense—by considering all possible combinations of those protonation states. While this type of computation would not be recommended for most prospective cases in the current form, future development of FEP and adjacent free energy methods to improve the handling of such coupled sites has the potential to reduce computational cost and may be extremely useful in subsequent design efforts.

The inclusion of additional structural data and analyses allowed us to gain some insight into the dynamics of the system and how domain-level motions may play a role in binding and function. The experimental crystal structures of *wt* Fc and three Fc variants in the unbound apo state over a range of pH values, together with analysis of these new structures and previously reported Fc structures, demonstrated a degree of flexibility in the relative domain orientation of the antibody Fc region that appeared to be pH-independent. In contrast, local conformations appeared to depend both on pH and on the specific amino acid sequence of the variant, suggesting that adoption of the holo conformation capable of binding to FcRn may also favor (or be favored by) certain mutations.

While the standard FEP protocol uses the same input structure for both the solvent and complex legs of the calculation, we found that using an apo Fc model specific to the unbound state yielded substantial improvement for a number of cases. However, not all cases improved, which may reflect differences in preferred global conformations for different mutations; we note that when using a single apo model for all mutations that we also assumed that the reorganization energy to adopt this conformation from the binding-competent conformation present in the complex structure is the same for all variants, which may not be the case in the physical system. With this in mind, we propose that a more complete understanding of the relative motion of Fc domains, in particular via the “breathing” or “twisting” normal modes, may be useful in Fc engineering and warrants further study.

With regard to the remaining outlier cases, we note that Fc variants M252H, M252R, and I253M are mutations of the loop containing the YTE mutations in the original crystal structure. Although we could not identify any obvious deficiencies in the all-atom *wt* model, it is possible that some subtle difference between the YTE crystal structure and the true *wt* conformation, inherited by our all-atom models, prevented sampling of a conformation relevant to the *wt* complex. Additionally, the only two other cases with errors larger than 2 kcal/mol were the Fc V259I/V308F/M428L (IFL) triple mutant, and the YTE Fc S254V variant. The IFL variant contained the M428L mutation from the LS variant, which is structurally near the location of the YTE Tyr252 sidechain, and S254V is also located on this particular loop. Further structural investigations of a human Fc-FcRn complex with a *wt* amino acid sequence for this loop may prove useful in determining whether these outliers may be due to structural differences in this loop region among the different variants in complex with FcRn.

We adopted an iterative approach to improve the agreement of computational predictions with experimental results, identifying potential deficiencies in our models and simulation protocols, and updating them based on new experimental and computational results. This methodology, similar to the typical Design-Make-Test-Analyze (DMTA) cycle used in drug discovery, may be just as readily applied in a prospective project as it is in the current retrospective study, with the understanding that a fraction of initial FEP predictions may exhibit significant errors due to incomplete sampling. The present work demonstrated the successful use of this iterative method by addressing outlier cases for which predicted ΔΔ*G* values deviated from experimental result, through further experimental studies and refinement of structural models and simulation parameters.

In summary, despite the challenges associated with predicting binding free energies at acidic pH for a system with a high density of titratable residues at the binding interface, including two histidines that are expected to undergo a change in protonation state as part of the pH-dependence mechanism, our FEP-based study was able to accurately reproduce experimental binding ΔΔ*G* values fora large dataset of single, double and triple mutant Fc-FcRn variants. By explicitly addressing the effects of coupled mutations on nearby titratable sites and accounting for conformational flexibility in unbound Fc, we were able to achieve a level of accuracy that has come to be expected from FEP results, and which is necessary for prospective work, thus opening the door for further efforts toward rational, *in silico* design of antibody Fcs with increased half-life.

## Materials and Methods

### Preparation of recombinant IgG, FcRn, and gp120 core for binding experiments

Expression vectors for Fc variants selected for experimental K_D_ measurement were generated by site-directed mutagenesis (GeneImmune) using the heavy chain expression vector for antibody VRC01 as a template [26]. Expression vectors for the recombinant soluble human FcRn α chain, the β2m chain with a C-terminal 8x His-tag, and clade A/E 93TH057 HIV-1 gp120 core(e) were constructed as described previously [28, 29]. Wild-type and variant Fc were expressed as IgG by transiently transfecting Expi293F cells (Thermo Fisher Scientific) with equal amounts of heavy and light chain expression plasmids using Turbo293 transfection reagent (SPEED Biosystem) according to manufacturers’ protocols. Wild-type and variant FcRn and gp120 core(e) were also produced in Expi293F cells by transient transfection. At 5 days post-transfection, cell culture supernatants were harvested and purified using Protein A Sepharose CL-4B resin (GE Healthcare) for Fc variants, cOmplete His-Tag purification resin (Sigma Aldrich) for FcRn and its variants, and 17b antibody-conjugated affinity column chromatography for gp120 core(e). The proteins were further purified by size-exclusion chromatography (SEC) using a HiLoad 16/600 Superdex 200 prep grade column (GE Healthcare).

### K_D_ measurement for IgG-FcRn binding by surface plasmon resonance

For SPR measurements, a CM5 sensor chip was coated with clade A/E 93TH057 HIV-1 gp120 core(e) immobilized by amine coupling and used to capture *wt* or Fc-variant VRC01 IgG to ~80-130 resonance units (RU) using Biacore T200 (GE Healthcare). Recombinant soluble *wt* or variant FcRn analytes were injected into the reference cell and the sample flow cell with the immobilized-IgG surface at concentrations ranging from 7.8 to 4000 nM or 7.8 to 10,000 nM in HBS-EP+ buffer (GE Healthcare) supplemented with 20 mM sodium citrate, pH 6.0, at a flow rate of 50 μL/min. Association and dissociation phases were monitored for 2 and 1 min, respectively. All SPR experiments were performed at 25°C. Reference-subtracted curves were fitted to a 1:1 Langmuir binding model or steady-state affinity model using Biacore T200 Evaluation Software 3.1.

### Calculation of ΔΔG from K_D_ values

We converted binding affinities (*K*_D_) to binding free energy (Δ*G*) values for each mutant using the standard relationship between dissociation constant and binding free energy, Δ*G* = –*RT*ln(*K*_D_/*C*), where R is the ideal gas constant (kcal mol^−1^ K^−1^), T is temperature (K), and C is the standard reference concentration of 1 M. Binding ΔΔ*G* values were computed for the variants in each publication by subtracting the *wt* Δ*G* from the Δ*G* of each corresponding mutant within the same study. For publications reporting measurements of the same Fc variant in different antibody (i.e. Fab) contexts, we reported a different ΔΔ*G* value for each unique variant-antibody combination, relative to the *wt* Fc measurement for the same antibody. ΔΔ*G* values of variants with measurements reported in multiple publications were averaged in the final dataset.

### Protein expression and purification for Fc crystallization

DNA sequences encoding *wt* IgG1 Fc, and M252H, M252R, and I253M Fc variant proteins were cloned into the mammalian expression vector pVRC8400. Point mutations were introduced into the constructs using KOD Hot Start polymerase (Novagen). The constructs were expressed in Expi293^™^ cells (Invitrogen), with polyethylenimine (Polysciences) used as the transfection reagent. Growth media were collected five days post-transfection, and secreted Fc proteins were purified using Protein A affinity chromatography, followed by SEC in PBS buffer at pH 7.4.

### Determination of apo Fc crystal structures

SEC fractions containing purified Fc proteins were pooled and concentrated to 10.5 mg/mL (*wt* Fc), 12.5 mg/mL (M252H Fc), 15.0 mg/mL (M252R Fc), or 9.0 mg/mL (I253M) in the SEC buffer. Screening for initial crystallization conditions was carried out in 96-well sitting drop plates using the vapor-diffusion method after setup with a Mosquito crystallization robot (TTP LabTech) using various commercially available crystallization screens: JSCG+ (Qiagen), MSCG-1 (Anthracene) and LMB and Proplex HT (Molecular Dimensions).

Diffraction quality crystals were obtained after 2 days under multiple conditions. For each variant, crystals from 2 or 3 crystallization conditions were selected for data collection, specifically including crystals that were grown at neutral/high pH or at acidic pH. For *wt* Fc: 0.1 M sodium citrate pH 5.5 (“WT low pH” structure, PDBID: 9CXL); and 0.1 M HEPES pH 7.0, 20% PEG 20,000, and 1 M ammonium sulfate (“WT high pH,” 9O7A). For M252H Fc: 0.1 M sodium citrate pH 5.6, 20% propanol, and 20% PEG 4000 (“M252H low pH,” 9D09); and 0.1 M Bis-tris propane pH 7.5 and 8% PEG8K (“M252H high pH,” 9CY6). For M252R Fc: 0.1 M sodium citrate pH 5.6, 20% propanol, and 20% PEG 4000 (“M252R low pH,” 9D06); and 0.1 M Tris pH 8.0, 0.08 M sodium formate, and 7.5% PEG 20,000 (“M252R high pH,” 9D9Q). For I253M Fc: 0.1 M citrate pH 5.5 and 15% PEG 6000 (“I253M pH 5.5,” 9O64); 0.1 M MES pH 6.5, 10% PEG 5000 and 12% propanol (“I253M pH 6.5,” 9O75); and 0.1 M MES pH 7.0, 15% PEG 20,000 and 12% propanol (“I253M pH 7.0,” PDBID: 9O78). Prior to data collection, crystals were cryoprotected in mother liquor supplemented with 40% ethylene glycol and flash frozen in liquid nitrogen.

X-ray diffraction data were collected at 100 K on beam line 17ID-1 (AMX) or 17 ID-2 (FMX) at the National Synchrotron Light Source II at Brookhaven National Lab (NY). Diffraction data were processed with autoPROC and scaled using AIMLESS from the CCP4 software suite [30, 31, 32]. Molecular replacement was performed with PHASER [33], using the Fc chain from PDB 6WNA as a search model [23]. Manual rebuilding of the structures using COOT [34] was alternated with refinement using Phenix.refine [35]. The MolProbity server [36] was used for structure validation and PyMOL was used for structure visualization and RMSD analysis [37].

### All-atom model preparation from crystal structures

Initial structural coordinates were obtained either from previously reported (YTE Fc-FcRn complex, PDB 6WNA) [23] or newly solved structures (apo Fc variants). For the complex structure, limited structural modeling and crystallographic refinement were performed using COOT [34] and Phenix [35], including the addition of ordered solvent near the binding interface. Bond orders were assigned and hydrogens added using the Protein Preparation Wizard panel (PPW) in Maestro (Schrödinger). Initial pKas were calculated and protonation states assigned within the PPW using PROPKA at pH 6.0 [38]. The YTE Fc chain from the 6WNA model was IgG4 subtype, and was monomeric due to 6 mutations disrupting the CH3-CH3 dimerization interface [23]. We mutated 17 residues in total to convert the model to the YTE IgG1 amino acid sequence. The required covalent bonds were modeled between sugar monomers, and any water molecules beyond 3 Å from the nearest protein atom were removed. Finally, a model of the *wt* Fc-FcRn complex was created from the YTE complex model by simple mutation of residues Y252, T254, and E256 in the YTE Fc chain to their *wt* counterparts, namely M252, S254, and T256, using the Maestro GUI. A more detailed accounting of all-atom model preparation is given in Supplementary Methods.

### FEP-based pKa calculation and pH-specific protonation state assignment

Protein FEP calculations were run using OPLS4 or OPLS5 to calculate titratable residue pKas as described previously [19]. Output perturbation map (.fmp) files and experimental pH (6.0) were used to determine pH-specific populations of each protonation microstate, variant Δ*G* values, and residue pKas following the Protein FEP Groups approach [22] via the protein_fep_groups.py script in the Schrödinger Suite. Protonation states in the input complex models were updated accordingly to create force field- and pH-specific models, with protonation states with the highest population in the bound complex state selected for each site.

### Retrospective FEP calculations

Protein FEP calculations were run for each of three data subsets (mutations of *wt* Fc, YTE Fc, or *wt* FcRn) with simulation times of 10 ns and OPLS5 force field using default protocols from the Schrödinger Suite 2024-2 release. Multiple mutations were performed sequentially according to the perturbation map automatically generated by the Protein FEP workflow. All alternate protonation states were included for perturbations to or from ionizable residues in the FEP map, and the Protein FEP Groups treatment was applied to calculate pH-specific predicted Δ*G* values for each variant at experimental pH 6.0. Although several measurements in the experimental dataset were actually reported using pH 5.8 as shown in Supplemental Figure 6, because FEP-predicted Δ*G* values after Protein FEP Groups treatment at pH 5.8 for those cases differed only negligibly from the pH 6.0 predicted Δ*G* values (by <0.1 kcal/mol in each case), only pH 6.0 predicted values are reported here. Likewise, the pH 5.8 experimental Δ*G* values were included as-is alongside corresponding pH 6.0 measurements when calculating average per-variant experimental Δ*G*s.

### Selection of variants for analysis of coupling to pH-sensor His residues

Putative pH-sensing Fc histidine residues H310 and H435 were selected for analysis to determine how their bound or unbound pKas and effect on overall predicted binding ΔΔ*G* were influenced by nearby mutations. Variants with potential coupled sites were identified for additional FEP calculations based on the following protocol. From the mutations.txt file used to prepare each FEP map (*wt* Fc, YTE Fc), “neighbor mutation sites” were selected which contained any sidechain atom within a given cutoff distance of any sidechain N or O atom in the coupling sites in the input all-atom model (in this case the relevant atoms were the H310 and H435 imidazole nitrogens). A cutoff of 6.0 Å was used for mutations where the neighboring residue was mutated to or from a charged or ionizable residue (D/E/H/K/R); a closer cutoff of 4 Å was used for neutral mutations. Variants containing any mutation at a selected site were included in separate FEP perturbation maps with all combinations of H310 and H435 protonation microstates, as well as the protonation microstates of ionizable residues at mutated sites (either *wt* or mutant), as described in Supplementary Methods.

### Calculation of solvent leg ΔG values using new crystal structure models

All-atom models of *wt* and YTE apo Fc were prepared starting from the crystal structure coordinates of the M252R low pH structure (PDB ID: 9D06). Residues 252, 254, and 256 in the Fc chain were mutated to either the *wt* (Met, Ser, Thr) or YTE (Tyr, Thr, Glu) sequences, respectively, to serve as input for subsequent apo-state FEP calculations. After preparation using the Maestro PPW as above, protonation states of all titratable residues were adjusted to match those in the pH 6.0 holo *wt* Fc model.

Solvent leg calculations for the retrospective set of mutations were performed using the *wt* or YTE apo Fc model using the same parameters used for the holo complex, except the calculation was run using the Protein FEP thermostability (rather than protein binding) protocol, as the FcRn binding partner was not present. The resulting solvent Δ*G* values were used to compute modified binding ΔΔ*G* values via the formula:

$$\text{ΔΔ}G_{\text{binding}}^{\text{mod.}}=\text{Δ}G_{\text{complex}}^{\text{holo}}-\text{Δ}G_{\text{solvent}}^{\text{apo}}\text{.}$$

### Normal mode analysis of Fc structures

Low frequency normal modes, describing collective atomic motions typically associated with the most flexible regions, were calculated for all monomeric holo and apo Fc structures as well as for a dimeric YTE Fc structure (prepared from PDB 4N0U via crystallographic symmetry) using a computationally efficient NOnLinear rigid Block normal mode analysis approach (NOLB) [39]. Normal modes for individual structures were computed using the command ./NOLB structure.pdb, and transitions between two structures using the two lowest-frequency modes were generated with the command ./NOLB structure1.pdb structure2.pdb -n 2 -s 20.

### Data analysis and figure and manuscript preparation

Data analysis was done using Python [40] and R [41]. Plots were generated in R using the ggplot2 package [42]. Structural figures were prepared using PyMOL [37]. The manuscript was prepared using Quarto and RStudio [43, 44].

## Supplementary Material

Supplement 1

2

## Figures and Tables

**Table 1.** FEP-calculated bound-state pKa values for interface residues in the 6WNA-derived models using OPLS4 or OPLS5.

		OPLS4		OPLS5		
Chain	Residue	WT	YTE	WT	YTE	Modeled^a^
Fc	T256 / E256	–	4.30	–	4.42	GLU
Fc	H285	6.58	6.55	6.68	6.63	HIP
Fc	H310	11.33	11.67	10.22	10.35	HIP
Fc	D312	2.88	2.84	2.92	3.11	ASP
Fc	E380	4.40	4.05	4.78	4.37	GLU
Fc	E430	5.56	5.86	6.13	6.47	GLU
Fc	H433	7.54	7.45	7.49	7.42	HIP
Fc	H435	6.17	5.70	6.96	6.42	HIP
FcRn	E115	−0.07	0.91	−0.22	1.79	GLU
FcRn	E116	2.45	2.54	3.19	3.28	GLU
FcRn	D130	4.05	4.00	4.12	3.93	ASP
FcRn	E133	2.04	3.30	2.63	2.82	GLU

a Protonation states selected for all-atom models used in retrospective FEP calculations.

**Table 2.** Summary of the experimental datasets used for FEP calculations.

	Number of Variants^a^				Experimental ΔΔ*G*^b^	
Subset	Single	Double	Triple	Total	Min	Max
WT Fc	34	18	11	63	−3.59	2.41
YTE Fc	11	3	2	16	−2.40	0.53
WT FcRn	5	0	0	5	0.15	1.86

Total	50	21	13	84	−3.59	2.41

a The number of single, double, and triple mutation variants, and total number of variants in each subset are shown.

b Experimental binding ΔΔ*G* range relative to the *wt* complex in kcal/mol.

## Data Availability

Experimental data, prepared all-atom structural models, and FEP results are available in the GitHub repository accompanying this manuscript, accessible via Zenodo [45] at DOI: 10.5281/zenodo.19777392.

## Significance

The ability to determine changes in binding affinity upon mutation is critical to structure-based protein design. In this study, we demonstrate a successful computational approach using free energy perturbation (FEP) calculations on the antibody Fc-FcRn complex, a medically relevant system with implications for both therapeutic and prophylactic antibody use. Our successful calculation of accurate binding energies across a wide range of cases speaks to the power of the FEP methodology in navigating the free energy landscapes of dynamic molecular complexes. Furthermore, we show that accurate Fc-FcRn affinity calculations required careful consideration of conformational flexibility between bound and unbound states, contributing to our functional understanding of a system that will be important for future rational antibody-design efforts.
