# Supplementary material for: A functional investigation of antibody Fc-FcRn variant binding guided by in silico free energy perturbation methods
Source: bioRxiv. 2026 Apr 30:2026.04.28.721095. Preprint. [Version 1] doi: 10.64898/2026.04.28.721095 (PMC13142375; doi:10.64898/2026.04.28.721095)
Supplement: 2 [file NIHPP2026.04.28.721095v1-supplement-2.pdf]

bioRxiv preprint doi: https://doi.org/10.64898/2026.04.28.721095; this version posted April 30, 2026. The copyright holder for this preprint (which was not certified by peer review) is the author/funder, who has granted bioRxiv a license to display the preprint in perpetuity. It is made available under aCC-BY 4.0 International license.

**Supplementary Information**

for

# A functional investigation of antibody Fc-FcRn variant binding guided by *in silico* free energy perturbation methods

Jared M. Sampson, Alina P. Sergeeva, Tianyang Gao, Young Do Kwon, Eswar Reddem, Fabiana A. Bahna, Seetha M. Mannepalli, Baoshan Zhang, Peter D. Kwong, Lawrence Shapiro, Barry Honig, Richard A. Friesner

---

## *Supplementary Methods*

### *All-atom Fc-FcRn structural model preparation and protonation state assignment*

We selected the X-ray crystal structure of a monomeric YTE variant human IgG4 Fc chain bound to the human FcRn+β2M heterodimer (PDB 6WNA, 2.4 Å resolution) as the starting point for our study [23]. We performed limited structural modeling and crystallographic refinement including the addition of visible ordered water molecules near titratable residues near the binding interface and an increase in the percentage of Ramachandran-favored residues from 95.48% to 98.58%. The final crystallographic model gave improved overall refinement statistics, reducing $R_{work}/R_{free}$ from 0.214/0.263 in the original PDB entry to 0.206/0.239 in the re-refined version.

We introduced 17 point mutations into the re-refined model in Maestro (Schrödinger, Inc.) to convert the Fc chain from IgG4 to the IgG1 subtype used for the experimental binding data. These included reversion of 6 mutations at the CH3-CH3 interface that had been introduced by Shan and coworkers to produce monomeric Fc [23]. Notably, none of these mutations involved residues at the FcRn binding interface. Finally, from this IgG1 YTE variant Fc-FcRn model, we made three mutations (Y252M/T254S/E256T) to revert the YTE residues to the *wt* sequence. The resulting united-atom *wt* and YTE variant IgG1 Fc-FcRn models were used as the basis for the preparation of all-atom models for use in FEP calculations.

Using the Protein Preparation Wizard in Maestro, we added hydrogens, assigned bond orders, and configured initial protonation states at pH 6.0 with PROPKA3 [38]. The resulting preliminary all-atom *wt* and YTE models were used as inputs to Protein FEP to calculate pKas for key interface titratable residues using either the OPLS4 or OPLS5 force fields, perturbing each interface titratable site from the initial modeled state to its alternate protonation states (e.g. `H:HIP310` to `HID` and `HIE`). The pKa values and the accompanying microstate populations reported after Protein FEP Groups treatment at pH 6.0 were used to determine pH-specific protonation states for each model according to which state was predicted to be dominant in

bioRxiv preprint doi: https://doi.org/10.64898/2026.04.28.721095; this version posted April 30, 2026. The copyright holder for this preprint (which was not certified by peer review) is the author/funder, who has granted bioRxiv a license to display the preprint in perpetuity. It is made available under aCC-BY 4.0 International license.

the bound complex. Interestingly, FEP-based pKa calculations using the fixed-charge OPLS4 force field predicted Fc residue H435 to be in the neutral `HID` tautomer in the bound state; however, the OPLS5 calculations for both *wt* and YTE models each yielded higher pKa values for H435, indicating the positively charged `HIP` microstate was preferred. As a final step, the indicated force field-specific protonation states were then incorporated into the models, resulting in the fully prepared pH 6.0 *wt* and YTE models ready for use in retrospective FEP calculations.

*Accounting for coupled titratable sites in FEP via explicit multiple mutations*

The Protein FEP Groups treatment was previously described in ref. [22] from the main text, and is based on a similar approach from Oliveira et al. [24]. For completeness, a brief summary of the methodology is included here, along with a description of how it can be used to treat coupling among titratable sites.

Protein FEP+ calculations involve alchemical transitions between fixed-topology models, so no protonation or deprotonation occurs over the course of the simulation in a time-dependent manner. However, by including FEP+ edges in the perturbation map in a way that provides alchemical perturbation paths from the microstate present in the starting model to all possible relevant protonation microstates for a given ionizable site or set of sites, from a single FEP+ map we can determine relative $\Delta\Delta G$s among all of the microstates, and therefore fractional populations of each microstate (as represented by a node in the FEP perturbation connectivity graph) in the unbound and bound physical macrostates at a given pH.

To account for protonation state effects on binding robustly, each perturbation requires three separate simulations, or “legs,” representing three physical macrostate contexts: bound state (complex leg); unbound state (solvent leg) and model state (fragment leg, run as a single, capped amino acid residue). From these simulations we obtain predicted $\Delta G$ values relative to the input structure for each microstate in each context. Using a single amino acid for the model leg allows the use of known pKas from the literature for the amino acids in this reference state to calculate model populations for each titratable site being considered using the Henderson-Hasselbalch equation (Equation 2):

$$\frac{[\text{protonated}]}{[\text{deprotonated}]} = 10^{\text{p}K_\text{a} - \text{pH}} \tag{2}$$

Given these literature-derived starting populations, if we consider the model, solvent, and complex as states 0, 1, and 2, respectively, the fractional solvent population ($p_i^1$) for each microstate $i$ can be calculated as a function of the model population ($p_i^0$) and the differences among the predicted folding free energies ($\Delta\Delta G_{i\to j}^{0\to 1}$) for all nodes $j$ (including $i$) in the same node group using Equation 3:

bioRxiv preprint doi: https://doi.org/10.64898/2026.04.28.721095; this version posted April 30, 2026. The copyright holder for this preprint (which was not certified by peer review) is the author/funder, who has granted bioRxiv a license to display the preprint in perpetuity. It is made available under aCC-BY 4.0 International license.

$$p_i^1 = \frac{1}{\sum_j \frac{p_j^0}{p_i^0} e^{-\Delta\Delta G_{i\to j}^{0\to 1}/kT}} \tag{3}$$

where $k$ is the Boltzmann constant and $T$ is the temperature in K. Similarly, the complex population for the node ($p_i^2$) can then be calculated from the solvent population using the same equation with the solvent populations and binding free energies as input (i.e. increment the indices for all superscripts).

With solvent ($p_i^1$) and complex ($p_i^2$) populations in hand, the overall pH-specific binding free energy of a given variant $X$ can be calculated by applying appropriate state penalties based on the fractional populations of the starting and ending states via:

$$\Delta G_X = \Delta G_i + kT \ln p_i^2 - kT \ln p_i^1 \tag{4}$$

For complex scenarios where multiple titratable sites must be considered, we treat the sites in one of two ways. First, if a titratable residue in the starting model is mutated to another amino acid in the perturbation map, it is perturbed to its alternate states in the `WT` node group, but this perturbation is not repeated in other variant node groups. In this case, the unbound and bound populations of that titratable residue in the *wt* context are applied as a factor to the unbound and bound populations of all variant node groups which retain that site. That is, the effects of titratable sites from the `WT` node group are inherited by the other variants if present, and assumed not to be affected by mutations in the variant. Furthermore, different sites in the `WT` node group are also assumed to be independent of one another, as a full combinatorial accounting of the microstates resulting from all titratable sites would be computationally intractable.

For specific cases, we employ a second approach, where coupling between titratable and/or mutation sites is explicitly defined by specifying a multiple mutation in the FEP+ input mutations file. For example, the mutation N434Y coupled to the pKa of *wt* titratable residue H435 would require having predicted Δ*G* values for the N434Y mutation in the context of each of the 3 His states at position 435. In the input `mutations.txt` file, with the `HIP435` protonation state in the starting model, this could be written as the following:

```
H:434->TYR
H:435->HID
H:435->HIE
H:434->TYR,H:435->HID
H:434->TYR,H:435->HIE
```

This input would result in 6 nodes total:

bioRxiv preprint doi: https://doi.org/10.64898/2026.04.28.721095; this version posted April 30, 2026. The copyright holder for this preprint (which was not certified by peer review) is the author/funder, who has granted bioRxiv a license to display the preprint in perpetuity. It is made available under aCC-BY 4.0 International license.

```
WT
H-HIP435HID
H-HIP435HIE
H-ASN434TYR
H-ASN434TYR,H-HIP435HID
H-ASN434TYR,H-HIP435HIE
```

with the first 3 nodes comprising the `WT` node group, and latter 3 nodes the `H-N434Y` node group. (Note that in the input mutations format, mutations separated by commas are automatically expanded by the FEP+ workflow to include all combinations of the component mutations, so although instructive here, the first 3 lines of the `mutations.txt` above are optional, as they are implied by the double-mutation lines.)

This process may also be applied to coupled titratable sites. For example, considering the interplay between the M252H mutation and *wt* Fc residues H310 and H435, both modeled as `HIP`, the mutations file used in our simulation included the following:

```
H:252->HID,H:310->HID,H:435->HID
H:252->HID,H:310->HID,H:435->HIE
H:252->HID,H:310->HIE,H:435->HID
H:252->HID,H:310->HIE,H:435->HIE
H:252->HIE,H:310->HID,H:435->HID
H:252->HIE,H:310->HID,H:435->HIE
H:252->HIE,H:310->HIE,H:435->HID
H:252->HIE,H:310->HIE,H:435->HIE
H:252->HIP,H:310->HID,H:435->HID
H:252->HIP,H:310->HID,H:435->HIE
H:252->HIP,H:310->HIE,H:435->HID
H:252->HIP,H:310->HIE,H:435->HIE
```

It should be noted that the simplifying assumption, that the perturbations sampled in FEP do not affect (and are not affected by) the protonation state of any other residue in the structure, is successful most of the time because the results produced by protein mutation FEP are *relative* free energy changes. Even if a protonation state (or change thereof) is important to binding, having an incorrect protonation state modeled will, in general, have minimal effect on most results because the calculations benefit from cancellation of error when both the *wt* and mutant simulations suffer from the same modeling error and the output result is reasonable. However, if the pKa of a nearby residue is tightly coupled to a perturbation (either a titration between microstates of a residue, or a mutation to a different amino acid), the errors are no longer balanced

bioRxiv preprint doi: https://doi.org/10.64898/2026.04.28.721095; this version posted April 30, 2026. The copyright holder for this preprint (which was not certified by peer review) is the author/funder, who has granted bioRxiv a license to display the preprint in perpetuity. It is made available under aCC-BY 4.0 International license.

and the effect on predicted Δ$G$ values can be substantial, as seen, for example, with the DHS variant discussed in the main text.

*Supplementary Results*

*Correction of the T307W outlier arising from convergence challenges*

The T307W mutation was moderately unfavorable according to SPR measurements (binding ΔΔ$G$ = 0.8 kcal/mol). Using the default FEP protocol—where the holo Fc conformation was employed in the solvent leg (see Figure 6)—we predicted a binding affinity change of -0.8 kcal/mol, resulting in an error of 1.6 kcal/mol. Notably, using the apo conformation in the solvent leg further increased the prediction error by approximately 1 kcal/mol. This result was unexpected, as the T307 residue is located in the CH2 domain and does not interact with the CH3 domain in either the holo or apo conformations (see Supplemental Figure 5A). Therefore, the choice of solvent leg conformation was not expected to significantly impact the prediction for this mutation, let alone exacerbate the error.

To investigate the anomalous behavior of the T307W mutation, we conducted three independent simulations using either the default or "extended" FEP protocol, that is, using either holo or apo conformation starting models in the solvent leg, respectively. Solvent leg energies exhibited substantial fluctuations in both cases, ranging from -4.76 to -6.33 kcal/mol for the holo conformation and from -3.97 to -4.77 kcal/mol for the apo conformation, indicating poor convergence across runs.

Since solvent leg energy was sensitive to the conformational states sampled, we examined pairwise interactions between T307W and nearby residues (within 6 Å) across all runs (Supplemental Table 5). The lowest solvent leg energy (-6.33 kcal/mol) was associated with stabilizing interactions: a hydrophobic contact between the methyl group of T256 and the indole ring of T307W (observed in 98% of the trajectory), a cation-pi interaction between K288 and the aromatic sidechain of T307W (98%), and a hydrogen bond between E258 and the N-H group of the T307W indole ring (65%). A representative structure is shown in the right panel of Supplemental Figure 5B. These sidechain interactions strongly correlated with more favorable solvent leg energies (Supplemental Table 5).

The local environment of the mutation site differed between the holo and apo structures (Supplemental Figure 5B). Residue T307 adopted distinct rotameric states in each, corresponding to the two most frequent rotamers found in crystal structures (46.8% in apo, 41.8% in holo). T256 also differed in orientation: in holo, it promoted a hydrophobic interaction with T307; in apo, its hydroxyl group instead faced T307, disrupting this contact. These differences contributed to less favorable solvent leg energies in the apo conformation, as reflected in the reduced frequency of T256-T307W interactions (Supplemental Table 5).

To confirm that local environment differences—and not interdomain angle—drove the increased error

bioRxiv preprint doi: https://doi.org/10.64898/2026.04.28.721095; this version posted April 30, 2026. The copyright holder for this preprint (which was not certified by peer review) is the author/funder, who has granted bioRxiv a license to display the preprint in perpetuity. It is made available under aCC-BY 4.0 International license.

with the apo structure, we ran reverse FEP simulations (Trp307→Thr) starting from mutant structures pre-stabilized in their local environment ("holo stab MT" and "apo stab MT"; Supplemental Figure 5C). Both simulations adequately sampled the stabilizing state and resulted in near-degenerate solvent leg energies, 5.38 and 5.61 kcal/mol, supporting the conclusion that the local environment was responsible for the discrepancy.

Next, we investigated whether one could improve sampling of the stabilizing state in the mutant trajectories by varying the rotamer state of the mutated residue. Three starting geometries were constructed for both holo and apo conformations, using the three most common Thr rotamers for T307. After manually selecting each rotamer, we minimized the mutated residue and its neighbors within 6 Å. Forward simulations of T307→W (Supplemental Table 6) revealed that only rotamer2 in holo conformation led to sampling of the stablizing state, yielding a solvent leg energy of -5.25 kcal/mol. Similarly, reverse simulations using various Trp rotamers yielded stabilizing interactions only in the apo rotamer2 case, with a corresponding energy of -5.46 kcal/mol. The range of solvent leg $\Delta G$ across rotamers 1-3 exceeded 1 kcal/mol for the T307→W mutation (Supplemental Table 6), likely due to the residue size change upon mutation. In contrast, mutations to residues closer in size to Thr (e.g. T307→Q,E) showed smaller solvent leg energy variations (0.5-0.6 kcal/mol), consistent with the stochastic range of FEP. Thus, undersampling was more pronounced for small-to-large mutations like T307W, and varying rotamers alone may be insufficient. One potentially more robust approach may involve sampling multiple rotameric starting points and selecting the mutant structure yielding the most favorable energy, assuming convergence in the *wt* runs.

Other mutations involving large sidechain size increases in our dataset—such as N434W and M252W—exhibited only modest solvent leg energy variation (0.2-0.3 kcal/mol) between holo and apo conformations Supplemental Table 7, suggesting the larger error seen for T307W was due to local structural sensitivity.

In this poorly converging case, we enhanced sampling through repeated simulations and identification of the most stable mutant state. Using the lowest solvent leg energies from three independent runs, we computed $\Delta\Delta G$ of the T307W mutant as $\Delta G$complex - $\Delta G$solvent = -6.01- (-6.33) = 0.32 kcal/mol, which reduces the prediction error from 1.6 to 0.5 kcal/mol, improving agreement with experiment ($\Delta\Delta G$ = 0.8 kcal/mol).

bioRxiv preprint doi: https://doi.org/10.64898/2026.04.28.721095; this version posted April 30, 2026. The copyright holder for this preprint (which was not certified by peer review) is the author/funder, who has granted bioRxiv a license to display the preprint in perpetuity. It is made available under aCC-BY 4.0 International license.

## *Supplementary Tables*

| Ligand: Fc variants | | Analyte: FcRn (7.8 - 4,000 nM) | | | | | | |
|---|---|---|---|---|---|---|---|---|
| | | Ligand (RU) | $K_D$ (nM)† | Std. Err. ($K_D$)† | $R_{max}$ (RU) | Indiv. Δ*G* | Mean Δ*G* | ΔΔ*G* from *wt* |
| Fc | **VRC01** | 84.2 | 1690 | 88 | 45.8 | -7.87 ± 0.03 | -7.99 ± 0.05 | 0.00 ± 0.07 |
| | **VRC01** | 97.1 | 1378 | 54 | 55.9 | -8.00 ± 0.02 | | |
| | **VRC01** | 73.9 | 1304 | 33 | 40.5 | -8.03 ± 0.01 | | |
| | **VRC01** | 82.0 | 1270 | 25 | 48.7 | -8.04 ± 0.01 | | |
| | **VRC01*** | 33.9 | 1390 | 64 | 20.6 | -7.99 ± 0.03 | | |
| | T250I | 108.5 | 1639 | 37 | 46.3 | -7.89 ± 0.01 | -7.89 ± 0.01 | 0.09 ± 0.05 |
| | L251N | 123.3 | 2281 | 63 | 46.5 | -7.70 ± 0.02 | -7.70 ± 0.02 | 0.29 ± 0.05 |
| | M252H | 118.2 | 5750 | 470 | 46.2 | -7.15 ± 0.05 | -7.15 ± 0.05 | 0.84 ± 0.07 |
| | M252R | 126.6 | 6053 | 220 | 56.6 | -7.12 ± 0.02 | -7.12 ± 0.02 | 0.87 ± 0.05 |
| | I253M | 111.3 | 4280 | 370 | 37.5 | -7.32 ± 0.05 | -7.32 ± 0.05 | 0.66 ± 0.07 |
| | S254C* | 118.6 | 78 μM | 120 μM | 81.3 | -5.61 ± 0.55 | -5.61 ± 0.55 | 2.38 ± 0.56 |
| | S254T | 132.1 | 2240 | 31 | 77.8 | -7.71 ± 0.01 | -7.71 ± 0.01 | 0.28 ± 0.05 |
| | S254V | 138.5 | 3050 | 39 | 77.2 | -7.52 ± 0.01 | -7.52 ± 0.01 | 0.46 ± 0.05 |
| | L309D | 69.8 | 5450 | 817 | 39.6 | -7.18 ± 0.08 | -7.18 ± 0.08 | 0.81 ± 0.1 |
| | Q311H | 107.1 | 1510 | 93 | 54.8 | -7.94 ± 0.04 | -7.94 ± 0.04 | 0.05 ± 0.06 |
| | Q311R | 136.8 | 861 | 41 | 73.2 | -8.27 ± 0.03 | -8.27 ± 0.03 | -0.29 ± 0.06 |
| | D312I | 126.2 | 2128 | 42 | 57.5 | -7.74 ± 0.01 | -7.74 ± 0.01 | 0.25 ± 0.05 |
| | D312S | 133.8 | 1120 | 9 | 67.4 | -8.12 ± 0.00 | -8.12 ± 0.00 | -0.13 ± 0.05 |
| | M428E | 118.3 | 4760 | 460 | 63.1 | -7.26 ± 0.05 | -7.26 ± 0.05 | 0.73 ± 0.07 |
| | M428L | 59.7 | 368 | 13 | 28.7 | -8.78 ± 0.02 | -8.78 ± 0.02 | -0.79 ± 0.05 |
| | L432K | 130.1 | 2172 | 50 | 59.9 | -7.73 ± 0.01 | -7.73 ± 0.01 | 0.26 ± 0.05 |
| | H433K | 81.2 | 872 | 31 | 44.3 | -8.27 ± 0.02 | -8.27 ± 0.02 | -0.28 ± 0.05 |
| | H433Q | 58.2 | 1310 | 17 | 31.8 | -8.03 ± 0.01 | -8.03 ± 0.01 | -0.04 ± 0.05 |
| | H433Y | 64.9 | 7070 | 270 | 37.3 | -7.03 ± 0.02 | -7.03 ± 0.02 | 0.96 ± 0.05 |
| | N434F | 169.4 | 98.9 | 3 | 90.4 | -9.56 ± 0.02 | -9.56 ± 0.02 | -1.57 ± 0.05 |
| | N434S | 56.5 | 294 | 8 | 30.3 | -8.91 ± 0.02 | -8.91 ± 0.02 | -0.92 ± 0.05 |
| | H435K | 64.7 | 386 | 6 | 35.9 | -8.75 ± 0.01 | -8.75 ± 0.01 | -0.76 ± 0.05 |
| | H435Q* | 35.5 | NBD | NBD | NBD | NBD | NBD | NBD |
| | H435Y* | 51.6 | 37 μM | 19 μM | 22.1 | -6.05 ± 0.25 | -6.05 ± 0.25 | 1.93 ± 0.25 |
| | M252Y/S254T | 93.5 | 245 | 3 | 55.2 | -9.02 ± 0.01 | -9.02 ± 0.01 | -1.03 ± 0.05 |
| | M252Y/T256E | 119.4 | 260 | 3 | 66.7 | -8.98 ± 0.01 | -8.98 ± 0.01 | -1.00 ± 0.05 |
| | S254T/T256E | 73.8 | 1108 | 35 | 36.2 | -8.12 ± 0.02 | -8.12 ± 0.02 | -0.14 ± 0.05 |
| | M428L/N434S | 115.3 | 117 | 3 | 64.1 | -9.46 ± 0.02 | -9.46 ± 0.02 | -1.47 ± 0.05 |
| | H433K/N434F | 125.8 | 72.0 | 3 | 66.2 | -9.74 ± 0.02 | -9.74 ± 0.02 | -1.76 ± 0.05 |
| | M252Y/S254C/T256E | 95.7 | 3230 | 210 | 24.7 | -7.49 ± 0.04 | -7.49 ± 0.04 | 0.50 ± 0.06 |
| | M252Y/S254V/T256E | 138.2 | 131 | 2 | 76.8 | -9.39 ± 0.01 | -9.39 ± 0.01 | -1.40 ± 0.05 |
| | L309D/Q311H/N434S | 90.4 | 312 | 7 | 68.3 | -8.88 ± 0.01 | -8.88 ± 0.01 | -0.89 ± 0.05 |
| | **YTE** | 63.3 | 169 | 5 | 35.2 | -9.24 ± 0.02 | -9.29 ± 0.02 | -1.30 ± 0.05 |
| | **YTE** | 51.2 | 142 | 4 | 27.8 | -9.34 ± 0.02 | | |
| | YTE + V308P | 141.2 | 26.1 | 2 | 89.1 | -10.34 ± 0.04 | -10.34 ± 0.04 | -2.36 ± 0.06 |
| | YTE + Q311H | 94.1 | 143 | 5 | 53.6 | -9.34 ± 0.02 | -9.34 ± 0.02 | -1.35 ± 0.05 |
| | YTE + A378V | 145.3 | 113 | 2 | 34.1 | -9.48 ± 0.01 | -9.48 ± 0.01 | -1.49 ± 0.05 |
| | YTE + H433K | 64.1 | 90.9 | 4 | 38.4 | -9.61 ± 0.03 | -9.61 ± 0.03 | -1.62 ± 0.06 |
| | YTE + N434F | 89.7 | 88.3 | 2 | 66.1 | -9.62 ± 0.01 | -9.62 ± 0.01 | -1.64 ± 0.05 |
| | YTE + N434S | 111.5 | 80.9 | 5 | 34.1 | -9.67 ± 0.03 | -9.67 ± 0.03 | -1.69 ± 0.06 |
| | YTE + Y436H | 63.2 | 276 | 35 | 36.3 | -8.95 ± 0.07 | -8.95 ± 0.07 | -0.96 ± 0.09 |
| **Analyte: FcRn variant (7.8 - 10,000 nM)** | | **Ligand: VRC01** | | | | | | |
| | | **Ligand (RU)** | **$K_D$ (nM)†** | **Std. Err. ($K_D$)†** | **$R_{max}$ (RU)** | **Indiv. Δ*G*** | **Mean Δ*G*** | **ΔΔ*G* from *wt*** |
| FcRn | FcRn-P132G | 119.5 | 6080 | 750 | 69.6 | -7.12 ± 0.07 | -7.12 ± 0.07 | 0.87 ± 0.09 |
| | FcRn-P132N | 115.4 | 16 μM | 1370 | 112.7 | -6.56 ± 0.05 | -6.56 ± 0.05 | 1.43 ± 0.07 |
| | FcRn-P132V | 117.9 | 1700 | 600 | 9.1 | -7.87 ± 0.18 | -7.87 ± 0.18 | 0.12 ± 0.19 |
| | FcRn-E133D | 120.0 | 1930 | 169 | 10.8 | -7.80 ± 0.05 | -7.80 ± 0.05 | 0.19 ± 0.07 |
| | FcRn-E133Q | 124.0 | 31 μM | 8000 | 174.3 | -6.16 ± 0.14 | -6.16 ± 0.14 | 1.83 ± 0.15 |
| | FcRn-wt | 123.6 | 1030 | 839 | 68 | -8.17 ± 0.35 | -8.17 ± 0.35 | -0.18 ± 0.36 |

* Analyte concentrations: 7.8 nM - 10,000 nM. † Concentrations are nM except where other units are specified.

Supplemental Table 1. Equilibrium $K_D$ and binding ΔΔ*G* values for *wt* and variant Fc-FcRn complexes at pH 6.0. Values for the binding of soluble FcRn analyte to immobilized VRC01 IgG from the measurements in Supplemental Figure 1. $K_D$ values and standard errors based on fits of steady-state responses or kinetic parameters over the indicated concentration series are indicated. Immobilized IgG ligand densities and estimated maximum response ($R_{max}$) are specified in resonance units (RU). Binding Δ*G*s are calculated for each measurement and averaged per variant, and resulting ΔΔ*G*s from the mean *wt* value are indicated.

bioRxiv preprint doi: https://doi.org/10.64898/2026.04.28.721095; this version posted April 30, 2026. The copyright holder for this preprint (which was not certified by peer review) is the author/funder, who has granted bioRxiv a license to display the preprint in perpetuity. It is made available under aCC-BY 4.0 International license.

| Variant | Expt. $\Delta\Delta G$ | Orig. Pred. $\Delta\Delta G$ | Coupled Pred. $\Delta\Delta G$ | $\Delta$ Abs. Err. |
|---|---|---|---|---|
| L251N | 0.32 | 0.62 | 0.64 | 0.02 |
| M252H | 0.87 | -1.40 | -1.95 | 0.55 |
| M252R | 0.90 | -2.08 | -1.92 | -0.16 |
| I253M | 0.69 | -2.43 | -1.79 | -0.64 |
| H435K | -0.73 | -2.50 | -1.79 | -0.71 |
| H435Y | 1.96 | 1.14 | 1.23 | -0.09 |
| L309D,Q311H,N434S | -0.91 | 2.78 | 1.01 | -1.77 |

Supplemental Table 2. Observed changes in predicted Binding $\Delta\Delta G$ values (kcal/mol) for 7 outlier cases before and after the inclusion of explicit coupling to Fc H310/H435 protonation states in FEP calculations.

bioRxiv preprint doi: https://doi.org/10.64898/2026.04.28.721095; this version posted April 30, 2026. The copyright holder for this preprint (which was not certified by peer review) is the author/funder, who has granted bioRxiv a license to display the preprint in perpetuity. It is made available under aCC-BY 4.0 International license.

| Apo Fc structure | WT low pH | WT high pH | M252H low pH | M252H high pH | M252R low pH | M252R high pH | I253M pH 5.5 | I253M pH 6.5 | I253M pH 7.0 |
|---|---|---|---|---|---|---|---|---|---|
| **Crystallization buffer pH** | 5.5 | 7 | 5.6 | 7.5 | 5.6 | 8 | 5.5 | 6.5 | 7 |
| **Wavelength** | 0.902 | 0.902 | 0.902 | 0.902 | 0.92 | 0.92 | 0.902 | 0.902 | 0.92 |
| **Resolution range** | 32.8-3.3 (3.85-3.37) | 33.5 - 2.1 (2.23-2.19) | 33.5 - 2.9 (3.68 - 2.92) | 68.7 - 2.0 (2.12 - 2.06) | 32.5 - 2.3 (2.39 - 2.33) | 45.8 - 2.82 (3.1 - 2.82) | 33.4 - 2.5 (2.76 - 2.6) | 41.8 - 2.5 (2.72 - 2.56) | 40.5 - 1.84 (1.89 - 1.85) |
| **Space group** | C 2 2 21 | P 21 21 21 | C 1 2 1 | P 21 21 21 | C 1 2 1 | P 21 21 21 | P 21 21 21 | P 21 21 21 | P 21 21 21 |
| **Unit cell** | 68.6 112.3 150.5 90 90 90 | 49.6 80.6 136.3 90 90 90 | 67.4 111.5 63.7 90 96.0 90 | 49.6 79.0 139.3 90 90 90 | 67.3 111.4 63.6 90 95.7 90 | 48.6 78.9 137.6 90 90 90 | 49.1 75.3 149.5 90 90 90 | 49.2 80.1 136.0 90 90 90 | 49.8 81.1 135.8 90 90 90 |
| **Total reflections** | 291361 (20818) | 13074 (3346) | 13352 (3156) | 15850 (9644) | 91671 (1191) | 182942 (4325) | 625480 (23242) | 625480 (89262) | 625480 (34708) |
| **Unique reflections** | 21555 (11540) | 7841 (5444) | 13018 (1013) | 8307 (6111) | 7589 (3326) | 15116 (1508) | 47933 (1729) | 47933 (6810) | 47933 (2776) |
| **Multiplicity** | 13.5 (13.5) | 1.7 (1.0) | 1.0 (1.0) | 1.9 (1.6) | 12.1 (7.3) | 12.1 (8.5) | 13.0 (13.4) | 13.0 (13.1) | 13.0 (12.5) |
| **Completeness (%)** | 99.3 (98.6) | 99.9 (99.7) | 72.9 (46.0) | 99.8 (98.7) | 99.0 (98.5) | 99.4 (98.4) | 96.1 (90.6) | 99.2 (98.0) | 99.9 (99.5) |
| **Mean I/sigma(I)** | 8.71 (-0.05) | 2.13 (2.86) | 4.11 (0.11) | 1.90 (1.12) | 6.44 (1.19) | 6.43 (0.80) | 6.55 (1.78) | 6.55 (1.28) | 6.55 (1.08) |
| **Wilson B-factor** | 45.2 | 44.8 | 49.9 | 44 | 59.1 | 73.6 | 30.7 | 57.8 | 26.3 |
| **R-merge** | 0.14 (8.949) | 0.47 (0) | 0.53 (0.74) | 0.53 (0.74) | 0.37 (0.85) | 0.37(2.6) | 0.25 (0.70) | 0.25 (0.9) | 0.25 (3.2) |
| **R-meas** | 0.15 (9.3) | 0.65 (0.80) | 0.53 (0.74) | 0.74 (1.0) | 0.38 (0.75) | 0.38 (2.8) | 0.26 (0.60) | 0.26 (3.0) | 0.26 (3.3) |
| **R-pim** | 0.04 (0.6) | 0.45 (0.650) | 0.45 (0.65) | 0.5(0.7) | 0.11(0.63) | 0.11 (0.95) | 0.07 (0.54) | 0.07 (0.84) | 0.074 (0.9) |
| **CC1/2** | 0.99 (0.05) | 0.621 (1) | 1 (1) | 0.28 (0.13) | 0.99 (0.68) | 0.99 (0.45) | 0.9 (0.6) | 0.99 (0.44) | 0.9 (0.3) |
| **CC*** | 1 (0.311) | 0.8 (1) | 1 (1) | 0.6 (0.4) | 0.9 (0.9) | 0.9 (0.7) | 0.99 (0.87) | 0.99 (0.78) | 0.9 (0.7) |
| **Reflections used in refinement** | 8489 (2774) | 28943 (1345) | 7427 (2325) | 34638 (2809) | 19846 (1385) | 13298 (3204) | 17090 (2613) | 17989 (2897) | 47910 (2763) |
| **Reflections used for R-free** | 383 (132) | 2876 (121) | 348 (136) | 1713 (162) | 1987 (138) | 619 (156) | 817 (102) | 898 (137) | 2352 (117) |
| **R-work** | 0.26 (0.34) | 0.22 (0.29) | 0.20 (0.26) | 0.23 (0.35) | 0.22 (0.31) | 0.28(0.34) | 0.28 (0.32) | 0.28 (0.32) | 0.21 (0.26) |
| **R-free** | 0.33 (0.36) | 0.28 (0.34) | 0.28(0.34) | 0.28(0.32) | 0.27 (0.31) | 0.36 (0.40) | 0.25 (0. 28) | 0.28 (0. 30) | 0.24 (0.28) |
| **Number of non-hydrogen atoms** | 2574 | 3678 | 2576 | 3737 | 2606 | 3284 | 2541 | 3641 | 4035 |
| **macromolecules** | 2492 | 3322 | 2494 | 3323 | 2495 | 3284 | 2541 | 3319 | 3327 |
| **ligands** | 82 | 220 | 82 | 220 | 82 | 0 | 0 | 220 | 220 |
| **solvent** | 0 | 136 | 0 | 194 | 29 | 0 | 0 | 102 | 488 |
| **Protein residues** | 312 | 414 | 312 | 414 | 312 | 408 | 318 | 414 | 414 |
| **RMS(bonds)** | 0.013 | 0.01 | 0.013 | 0.01 | 0.01 | 0.01 | 0.009 | 0.009 | 0.007 |
| **RMS(angles)** | 1.65 | 1.17 | 1.25 | 1.17 | 1.17 | 1.21 | 1.15 | 1.12 | 0.93 |
| **Ramachandran favored (%)** | 89.61 | 95.85 | 94.16 | 97.32 | 97.4 | 87.13 | 90.44 | 94.39 | 98.04 |
| **Ramachandran allowed (%)** | 10.3 | 4.1 | 5.8 | 2.6 | 2.6 | 12.8 | 9.5 | 5.6 | 1.95 |
| **Ramachandran outliers (%)** | 0 | 0 | 0 | 0 | 0 | 0 | 0 | 0 | 0 |
| **Rotamer outliers (%)** | 1.72 | 3.35 | 7.59 | 4.13 | 6.55 | 8.38 | 9.12 | 3.88 | 1.03 |
| **Clashscore** | 9.85 | 6.3 | 8.44 | 8.6 | 7.3 | 4.13 | 5.35 | 3.62 | 7.87 |
| **PDBID** | **9CXL** | **9O7A** | **9D09** | **9CY6** | **9D06** | **9D9Q** | **9O64** | **9O75** | **9O78** |

Supplemental Table 3. Crystallographic data collection and refinement statistics for apo Fc structures. All structures were solved by molecular replacement. Values in parentheses are for the highest resolution shell. Crystallization buffer pH and PDB Accession numbers are also shown.

bioRxiv preprint doi: https://doi.org/10.64898/2026.04.28.721095; this version posted April 30, 2026. The copyright holder for this preprint (which was not certified by peer review) is the author/funder, who has granted bioRxiv a license to display the preprint in perpetuity. It is made available under aCC-BY 4.0 International license.

| Reference structure | WT low pH (apo) | WT high pH (apo) | WT (holo) |
|---|---|---|---|
| WT low pH (apo) | - | (0.6, 0.5, 17.7, 4.6) | (0.6, 0.7, 14.8, 4.4) |
| M252H low pH (apo) | (0.4, 0.4, 2.3, 0.6) | (0.4, 0.4, 20.4, 5.6) | (0.4, 0.7, 16.8, 4.8) |
| M252R low pH (apo) | (0.4, 0.4, 2.4, 0.6) | (0.4, 0.3, 20.7, 6.0) | (0.4, 0.7, 16.8, 4.8) |
| WT high pH (apo) | (0.6, 0.5, 17.7, 4.6) | - | (0.4, 0.6, 10.1, 2.8) |
| M252H high pH (apo) | (0.6, 0.4, 17.6, 4.5) | (0.1, 0.2, 0.9, 0.4) | (0.4, 0.7, 9.0, 2.4) |
| M252R high pH (apo) | (0.6, 0.5, 12.8, 4.2) | (0.7, 0.3, 10.9, 4.2) | (0.5, 0.7, 2.1, 1.1) |
| WT holo (apo) | (0.6, 0.7, 14.8, 4.4) | (0.4, 0.6, 10.1, 2.8) | - |
| I253M pH=5.5 (apo) | (0.7, 0.6, 15.3, 3.7) | (0.4, 0.4, 4.7, 1.2) | (0.6, 0.7, 9.1, 2.1) |
| I253M pH=6.5 (apo) | (0.6, 0.5, 16.9, 4.2) | (0.2, 0.2, 0.9, 0.2) | (0.5, 0.7, 9.4, 2.8) |
| I253M pH=7.0 (apo) | (0.6, 0.4, 17.9, 4.6) | (0.1, 0.1, 0.5, 0.1) | (0.4, 0.6, 9.9, 2.8) |

Supplemental Table 4. Comparison of crystallographically solved structures from this study with three reference *wt* conformations. Structural differences are assessed using four metrics: RMSD', RMSD", angle, and displacement (detailed below). The structures (rows) are superimposed on the CH2 domain of the reference structures (columns). RMSD' represents the root-mean-square deviation (RMSD) between CH2 domains, reflecting their structural similarity. From this aligned position, CH3 domain reorientation is quantified using displacement (translation in Å) and angle (rotation in °). RMSD" measures the RMSD between CH3 domains, capturing conformational divergence in this region. The reorientation of the CH3 domain was quantified using the https://pymolwiki.org/index.php/Angle_between_domains script in PyMOL.

bioRxiv preprint doi: https://doi.org/10.64898/2026.04.28.721095; this version posted April 30, 2026. The copyright holder for this preprint (which was not certified by peer review) is the author/funder, who has granted bioRxiv a license to display the preprint in perpetuity. It is made available under aCC-BY 4.0 International license.

| **ΔG holo solvent** | **E258-T307W ss Hbond** | E258-T307W water bridge | **K288-T307W cation-pi** | K288-T307W hydrophobic | **T256-T307W hydrophobic** | **T256-T307W ss Hbond** | V305-T307W hydrophobic | N286-T307W water bridge | N286-T307W hydrophobic |
|---|---|---|---|---|---|---|---|---|---|
| repeat1 (-4.76) | 55.81% | 6.98% | 51.16% | 51.16% | 79.07% | 0.00% | 32.56% | 74.42% | 27.91% |
| repeat2 (**-6.33**) | **65.12%** | 4.65% | **97.67%** | 20.93% | **97.67%** | 0.00% | 0.00% | 88.37% | 0.00% |
| repeat3 (-5.65) | 51.16% | 13.95% | 81.40% | 27.91% | 88.37% | 0.00% | 9.30% | 69.77% | 4.65% |

| **ΔG apo solvent** | **E258-T307W ss Hbond** | E258-T307W water bridge | **K288-T307W cation-pi** | K288-T307W hydrophobic | **T256-T307W hydrophobic** | **T256-T307W ss Hbond** | V305-T307W hydrophobic | N286-T307W water bridge | N286-T307W hydrophobic |
|---|---|---|---|---|---|---|---|---|---|
| repeat1 (-3.97) | 41.86% | 4.65% | 74.42% | 20.93% | 44.19% | 4.65% | 0.00% | 79.07% | 4.65% |
| repeat2 (-4.77) | 44.19% | 4.65% | 32.56% | 93.02% | 32.56% | 0.00% | 72.09% | 60.47% | 9.30% |
| repeat3 (-4.71) | 44.19% | 16.28% | 30.23% | 81.40% | 27.91% | 2.33% | 48.84% | 41.86% | 25.58% |

Supplemental Table 5. Analysis of pairwise chemical interactions between the T307W residue and its neighboring residues in the solvent leg trajectories. To estimate the Δ*G* of the solvent leg, three independent 10 ns simulations were performed using the OPLS5 force field and different random seeds. The percent occurrence of each interaction was calculated using the `analyze_trajectory_ppi.py` script from the Schrödinger suite. Key interactions contributing to solvent leg energy differences (described in Supplemental Figure 5B, right panel) are highlighted in bold text.

| **Forward mutation** | apo rotamer1 | apo rotamer2 | apo rotamer3 | holo rotamer1 | holo rotamer2 | holo rotamer3 | Range |
|---|---|---|---|---|---|---|---|
| THR307TRP | -4.81 | -4.58 | -4.74 | -4.07 | **-5.25** | -4.07 | 1.18 |
| THR307GLN | -48.70 | -48.78 | -49.23 | -48.79 | -49.06 | -49.01 | 0.53 |
| THR307GLU | -156.02 | -155.81 | -155.63 | -156.19 | -155.81 | -156.07 | 0.56 |
| THR307GLH | -36.86 | -36.91 | -36.98 | -37.04 | -37.29 | -36.75 | 0.54 |

| **Reverse mutation** | holo stab | holo rotamer1 | holo rotamer2 | holo rotamer3 |
|---|---|---|---|---|
| TRP307THR | **5.38** | 5.07 | 4.78 | 4.81 |
| | apo stab | apo rotamer1 | apo rotamer2 | apo rotamer3 |
| | **5.61** | 4.47 | 5.46 | 4.55 |

Supplemental Table 6. Dependence of solvent leg FEP energies on variations in the local environment near the mutation site in the input structures. Δ*G* values for the solvent leg were calculated from 10 ns simulations using the OPLS5 force field. Forward FEP simulations (Thr307 → Trp, Gln, neutral or charged Glu) were initiated from one of the three most common rotameric states of Thr in either the apo or holo conformation. Reverse simulations (Trp307 → Thr) used input geometries shown in Supplemental Figure 5C.

bioRxiv preprint doi: https://doi.org/10.64898/2026.04.28.721095; this version posted April 30, 2026. The copyright holder for this preprint (which was not certified by peer review) is the author/funder, who has granted bioRxiv a license to display the preprint in perpetuity. It is made available under aCC-BY 4.0 International license.

**M252W**

| repeat | complex Δ*G* holo | solvent Δ*G* holo | solvent Δ*G* apo |
|---|---|---|---|
| 1 | 6.29 | 4.84 | 5.08 |
| 2 | 6.00 | 4.73 | 5.56 |
| 3 | 5.16 | 5.10 | 4.88 |
| min | 5.16 | 4.73 | 4.88 |
| mean | 5.82 | 4.89 | 5.17 |
| stdev | 0.59 | 0.19 | 0.35 |

| | |
|---|---|
| Solvent leg correction, apo – holo (min) | -0.15 |
| Solvent leg correction, apo – holo (mean) | -0.28 |
| Mean ΔΔG (holo solvent) | 0.43 |
| Mean ΔΔG (apo solvent) | 0.28 |
| Expt. ΔΔG | -1.07 |
| Absolute error (holo solvent) | 1.5 |
| Absolute error (apo solvent) | 1.35 |

**T307W**

| repeat | complex Δ*G* holo | solvent Δ*G* holo | solvent Δ*G* apo |
|---|---|---|---|
| 1 | -5.94 | -4.76 | -3.97 |
| 2 | -5.16 | -6.33 | -4.77 |
| 3 | -6.01 | -5.65 | -4.71 |
| min | -6.01 | -6.33 | -4.77 |
| mean | -5.70 | -5.58 | -4.48 |
| stdev | 0.47 | 0.79 | 0.45 |

| | |
|---|---|
| Solvent leg correction, apo – holo (min) | -1.56 |
| Solvent leg correction, apo – holo (mean) | -1.10 |
| Mean ΔΔG (holo solvent) | 0.32 |
| Mean ΔΔG (apo solvent) | -1.24 |
| Expt. ΔΔG | 0.80 |
| Absolute error (holo solvent) | 0.48 |
| Absolute error (apo solvent) | 2.04 |

**N434W**

| repeat | complex Δ*G* holo | solvent Δ*G* holo | solvent Δ*G* apo |
|---|---|---|---|
| 1 | 38.56 | 40.78 | 40.89 |
| 2 | 38.57 | 41.29 | 41.32 |
| 3 | 38.80 | 40.77 | 41.36 |
| min | 38.56 | 40.77 | 40.89 |
| mean | 38.64 | 40.95 | 41.19 |
| stdev | 0.14 | 0.30 | 0.26 |

| | |
|---|---|
| Solvent leg correction, apo – holo (min) | -0.12 |
| Solvent leg correction, apo – holo (mean) | -0.24 |
| Mean ΔΔG (holo solvent) | -2.21 |
| Mean ΔΔG (apo solvent) | -2.33 |
| Expt. ΔΔG | -2.20 |
| Absolute error (holo solvent) | 0.01 |
| Absolute error (apo solvent) | 0.13 |

Supplemental Table 7. Comparison of solvent leg energies using holo and apo conformations, from 10 ns Protein FEP simulations using OPLS5 force field; all Δ*G*/ΔΔ*G* values in kcal/mol.

bioRxiv preprint doi: https://doi.org/10.64898/2026.04.28.721095; this version posted April 30, 2026. The copyright holder for this preprint (which was not certified by peer review) is the author/funder, who has granted bioRxiv a license to display the preprint in perpetuity. It is made available under aCC-BY 4.0 International license.

*Supplementary Figures*

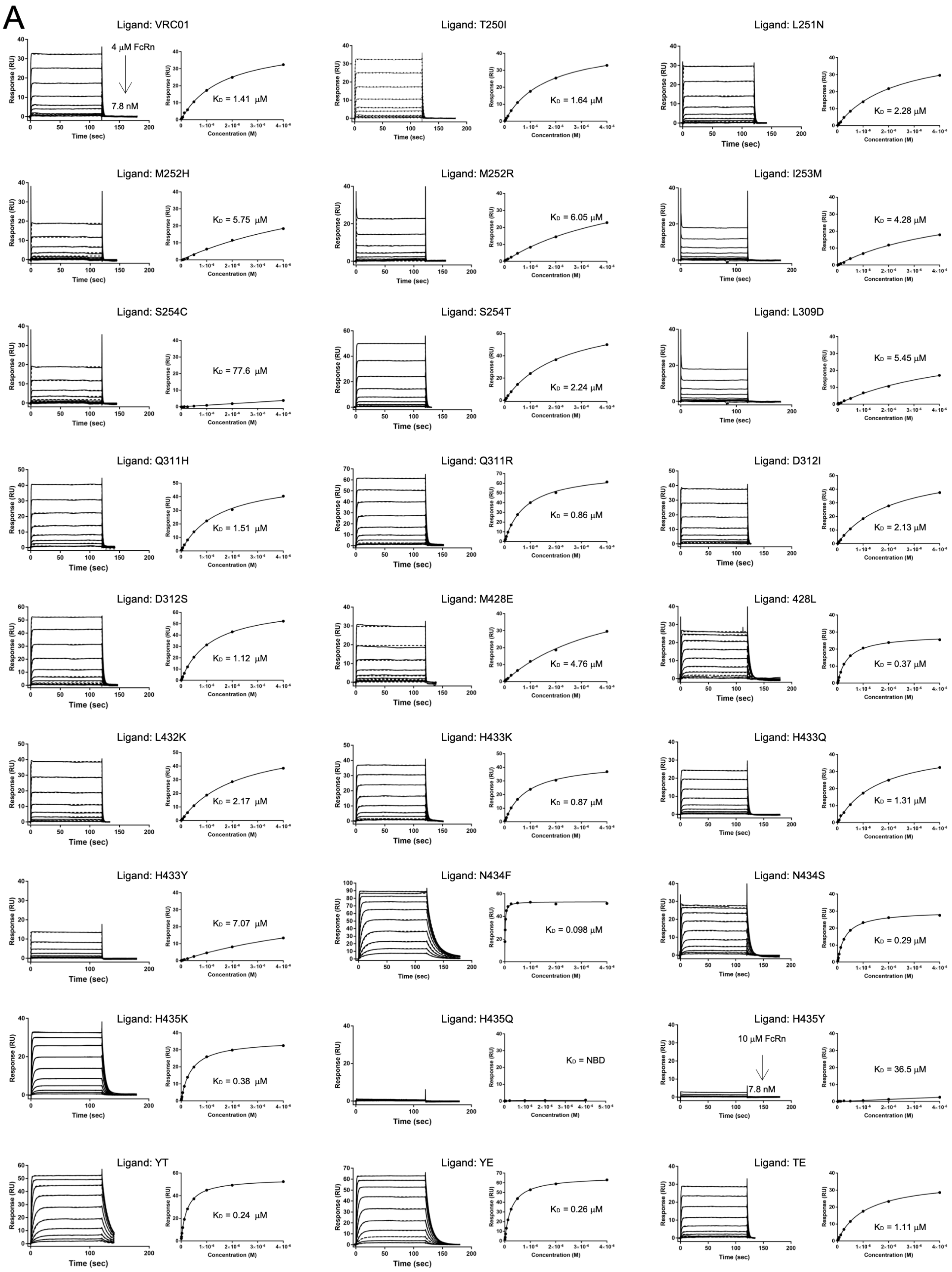

bioRxiv preprint doi: https://doi.org/10.64898/2026.04.28.721095; this version posted April 30, 2026. The copyright holder for this preprint (which was not certified by peer review) is the author/funder, who has granted bioRxiv a license to display the preprint in perpetuity. It is made available under aCC-BY 4.0 International license.

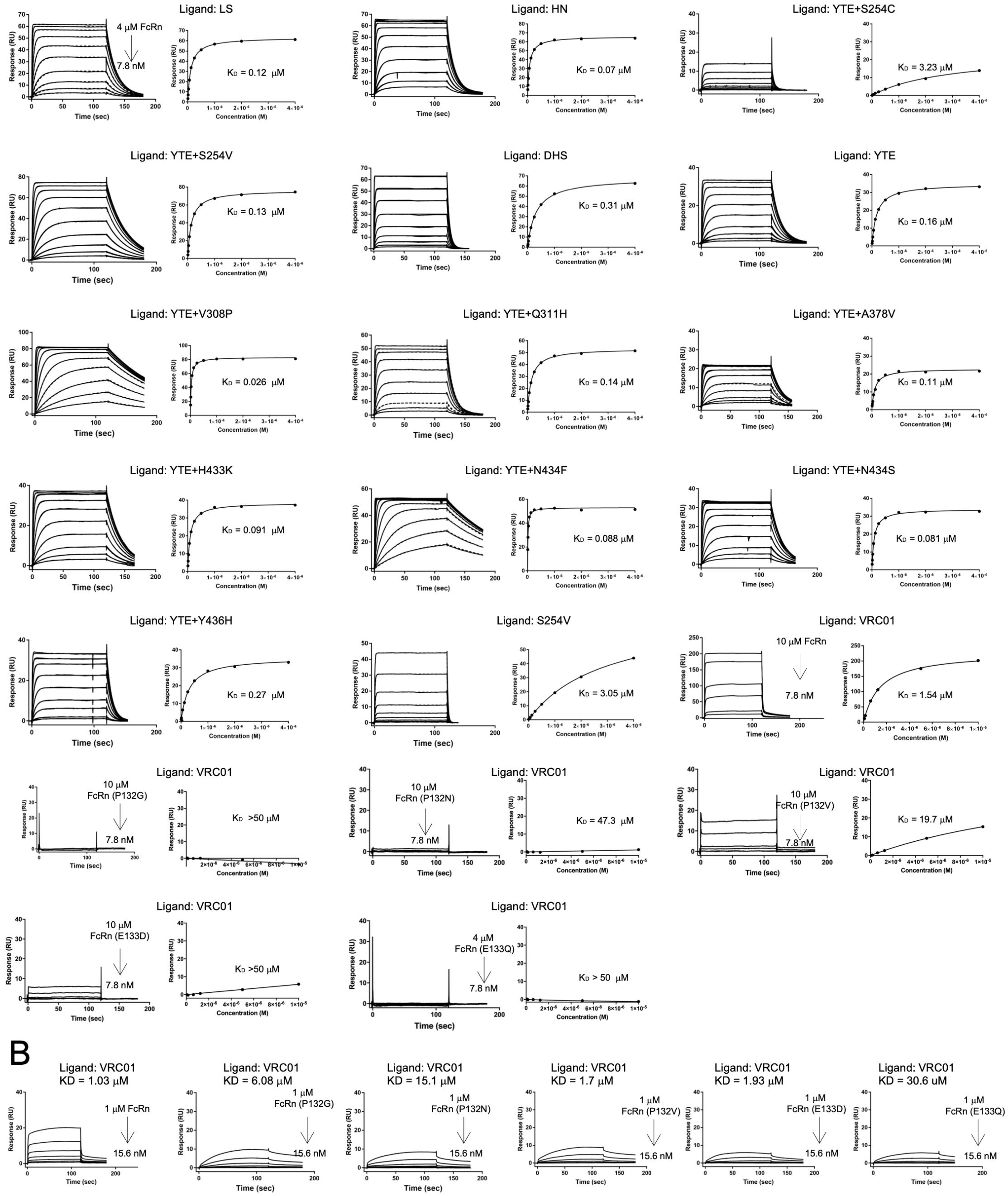


Supplemental Figure 1. SPR binding measurements for Fc-FcRn variants at pH 6.0. (A) *Left panels:* Binding sensorgrams for concentration series of *wt* FcRn analyte injected over immobilized Fc-variant VRC01 ligands. *Right panels:* Equilibrium binding responses plotted on a linear scale against injected FcRn variant concentrations and fit to a simple binding isotherm. Inset $K_D$ values correspond to the FcRn concentration that generated 0.5 $R_{max}$. (B) Binding sensorgrams for variant FcRn analytes injected over immobilized *wt* VRC01 ligand, and fit to a 1:1 Langmuir binding model. Inset $K_D$ values correspond to the ratio between dissociation and association rate constants, $k_{\text{off}}/k_{\text{on}}$.

bioRxiv preprint doi: https://doi.org/10.64898/2026.04.28.721095; this version posted April 30, 2026. The copyright holder for this preprint (which was not certified by peer review) is the author/funder, who has granted bioRxiv a license to display the preprint in perpetuity. It is made available under aCC-BY 4.0 International license.

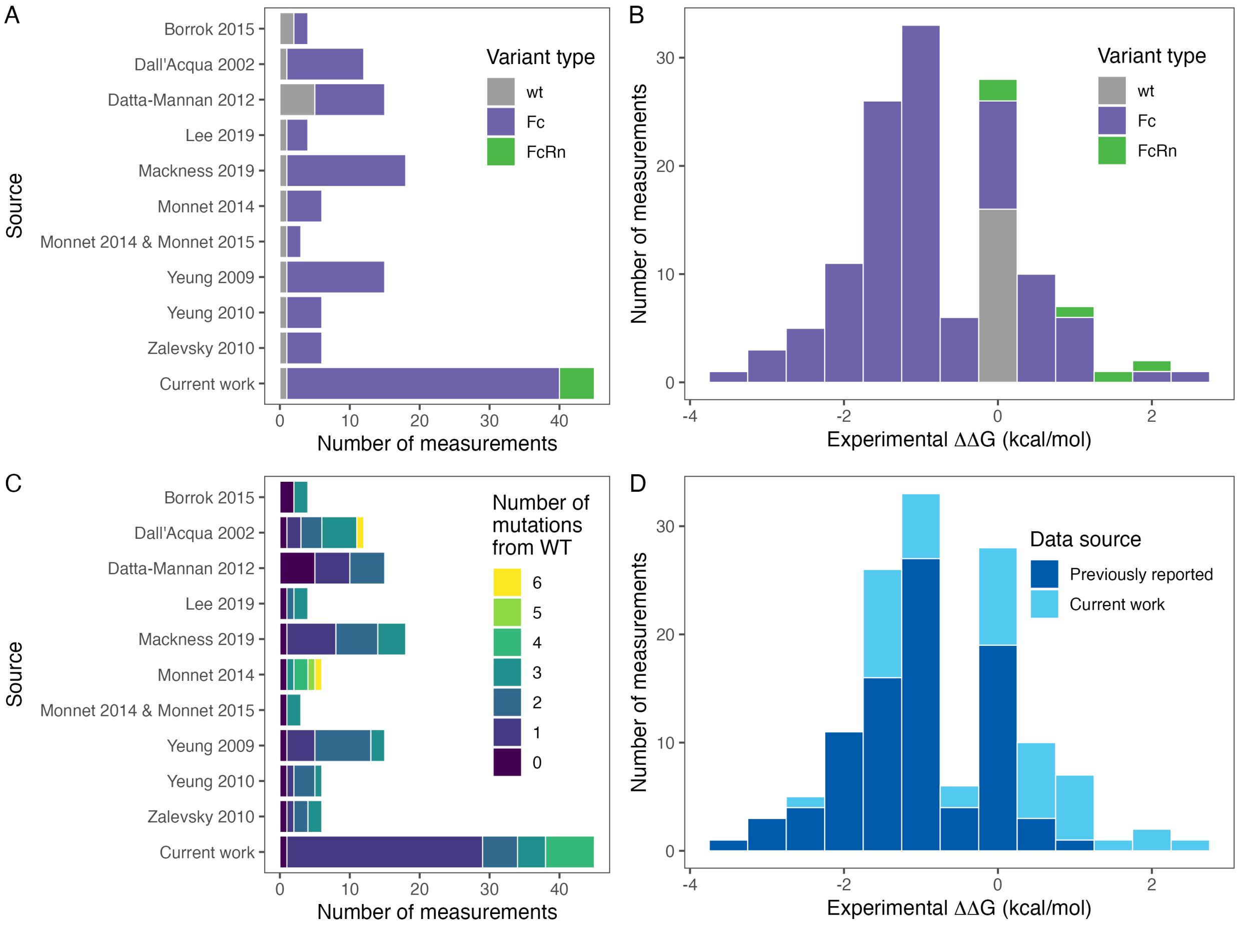


Supplemental Figure 2. Histograms providing additional information about the experimental dataset. (A) Number of experimental Fc-FcRn variant binding affinity measurements obtained from each published source and the current work, colored by mutated protein. (B) Distribution of experimental binding free energies (Δ$\Delta G$) of the full dataset relative to the *wt* Fc-FcRn complex, colored as in (A). (C) Breakdown of the number of mutations from *wt* Fc-FcRn present in the variants from each source. (D) Distribution of experimental $\Delta\Delta G$s, showing increased dynamic range and improved coverage of postive $\Delta\Delta G$ values after including the measurements from the current work.

bioRxiv preprint doi: https://doi.org/10.64898/2026.04.28.721095; this version posted April 30, 2026. The copyright holder for this preprint (which was not certified by peer review) is the author/funder, who has granted bioRxiv a license to display the preprint in perpetuity. It is made available under aCC-BY 4.0 International license.

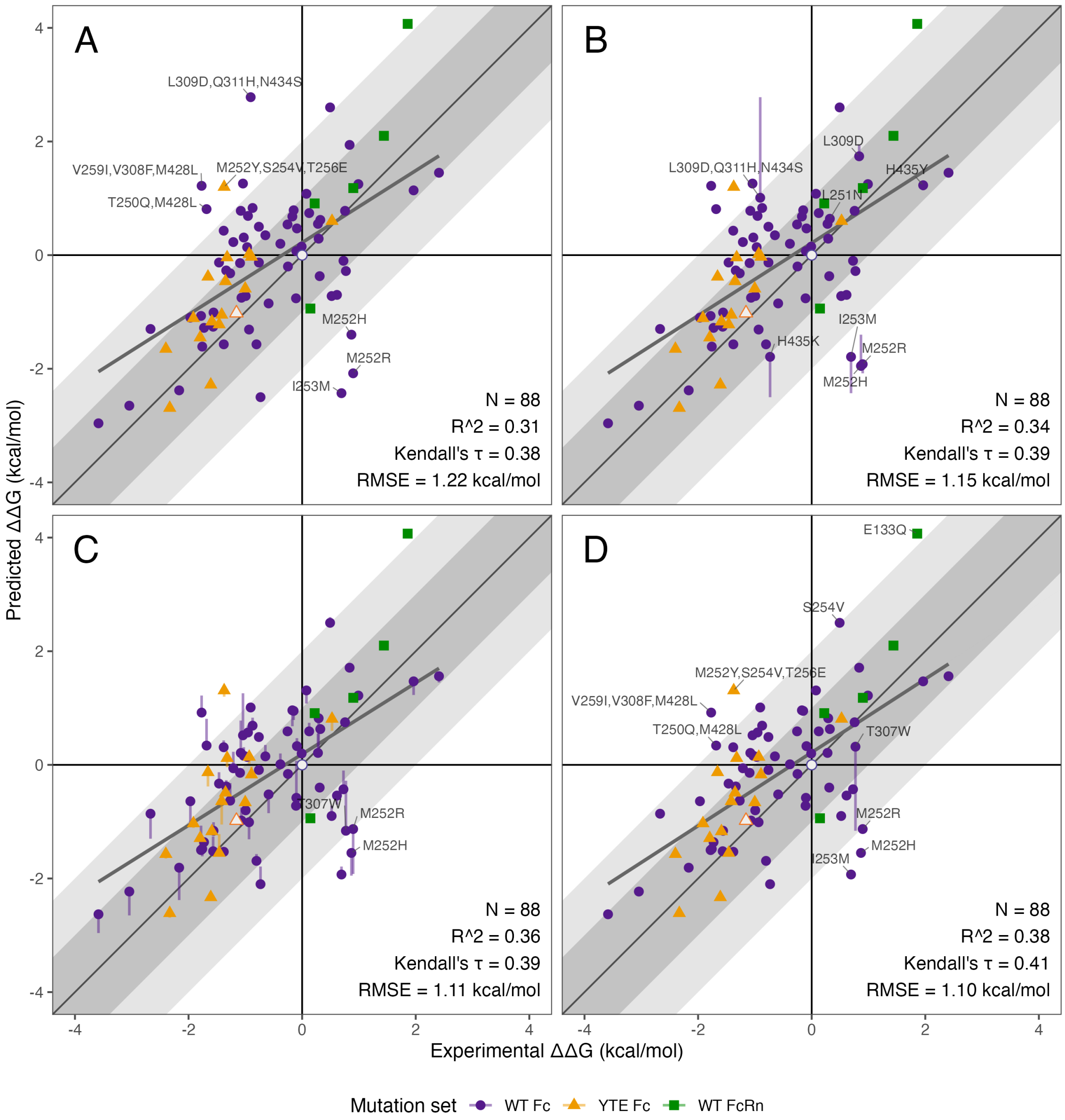


Supplemental Figure 3. Intermediate retrospective FEP results. Results are shown from 10 ns FEP simulations at four stages: (A) original results using OPLS5 force field and default protocols; (B) accounting for coupling between mutation sites and key titratable Fc His residues H310 and H435; (C) incorporating apo Fc solvent leg $\Delta G$ values; and (D) addressing a local conformational difference in the apo model for the Fc T307W variant. Vertical tails indicate change in predicted $\Delta\Delta G$ from the previous stage.

bioRxiv preprint doi: https://doi.org/10.64898/2026.04.28.721095; this version posted April 30, 2026. The copyright holder for this preprint (which was not certified by peer review) is the author/funder, who has granted bioRxiv a license to display the preprint in perpetuity. It is made available under aCC-BY 4.0 International license.

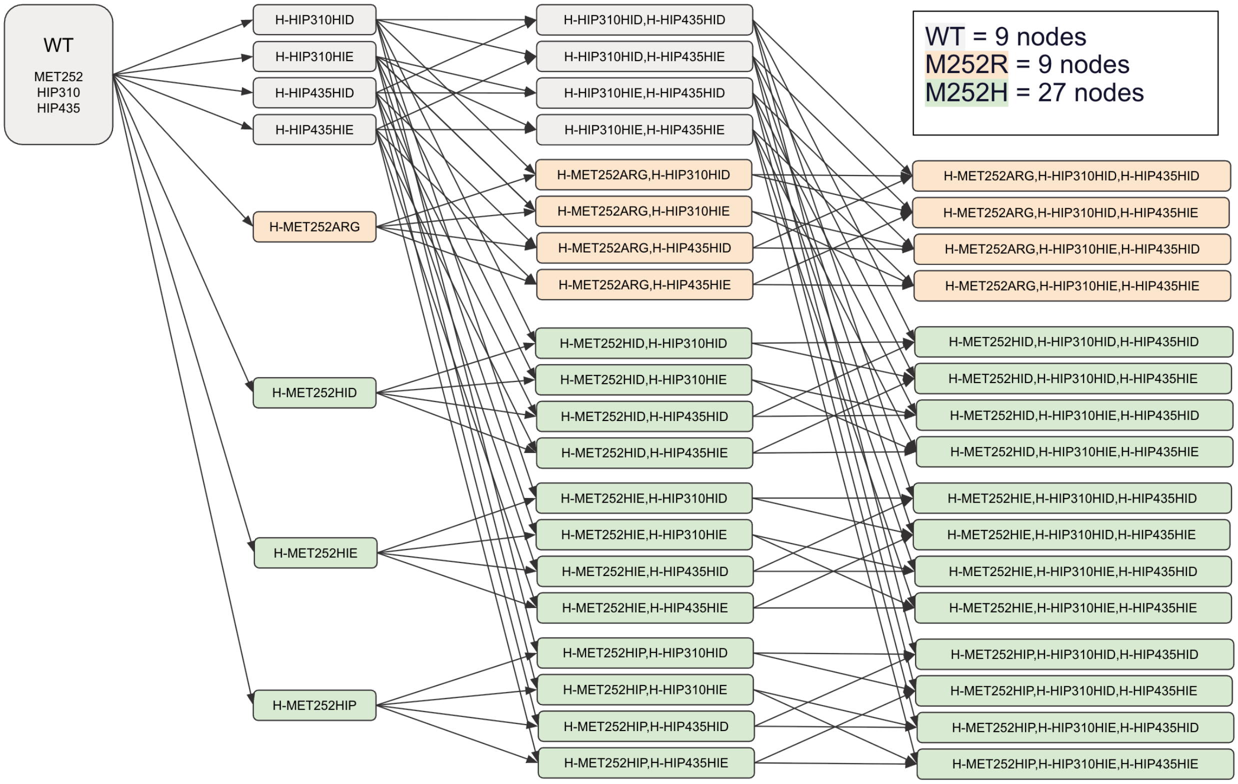


Supplemental Figure 4. Example illustration of Protein FEP graph topology for Fc M252R and M252H variants with explicit coupled titration of Fc H310 and H435.

bioRxiv preprint doi: https://doi.org/10.64898/2026.04.28.721095; this version posted April 30, 2026. The copyright holder for this preprint (which was not certified by peer review) is the author/funder, who has granted bioRxiv a license to display the preprint in perpetuity. It is made available under aCC-BY 4.0 International license.

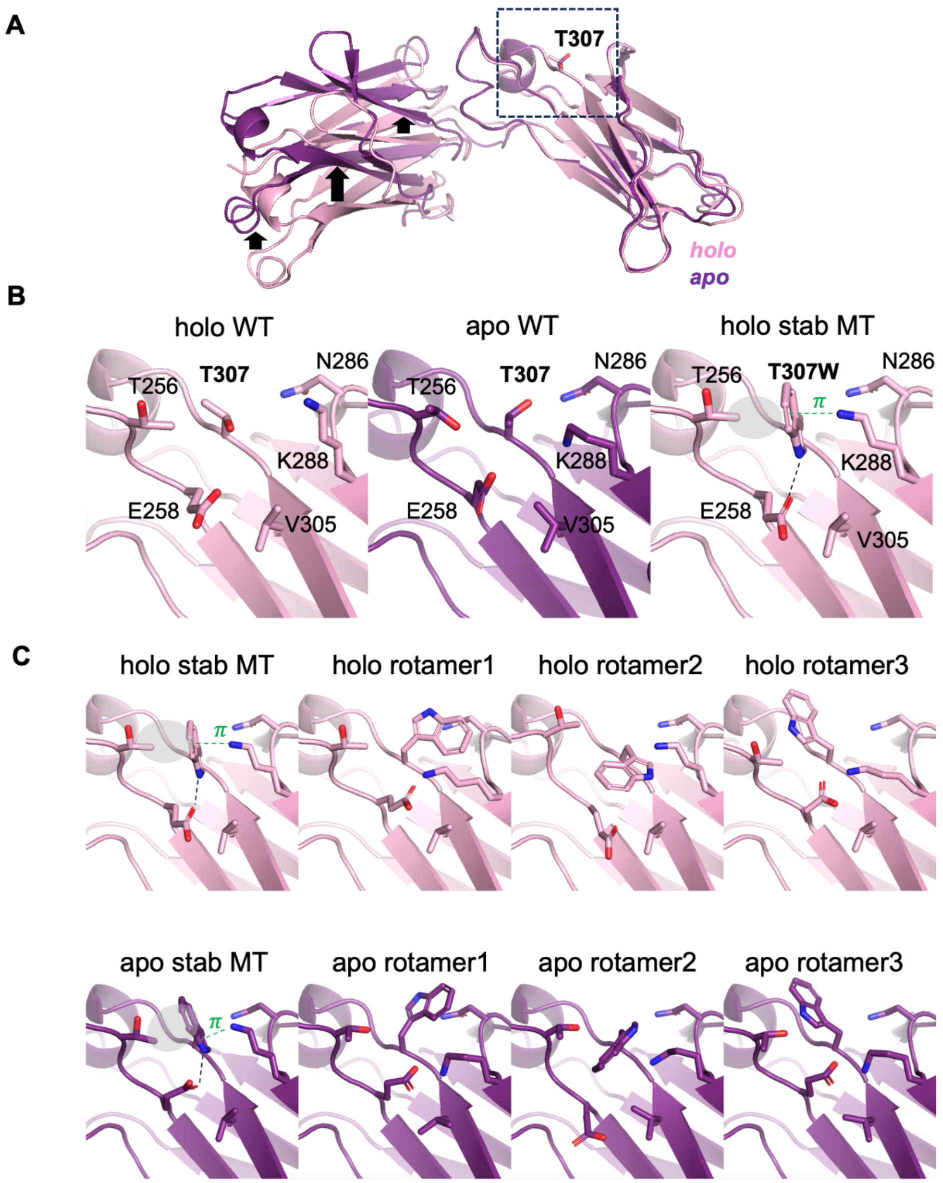


Supplemental Figure 5. Structural heterogeneity contributing to convergence issues in the T307W outlier. (A) Ribbon representation of holo (pink) and apo (purple) Fc conformations aligned on the CH2 domain, with the mutation site indicated by a dashed box, far from the region undergoing conformational change. (B) Close-up view of the boxed region in (A), showing the side chain of residue 307 and surrounding amino acids in stick representation. The stable interaction network observed in simulations with low energy of the mutant state (right panel) includes a hydrophobic contact between T256 and T307W (gray oval), a cation-π interaction between K288 and T307W (green), and a hydrogen bond between E258 and T307W. (C) Input structures used for reverse FEP simulations (Trp307→Thr).

bioRxiv preprint doi: https://doi.org/10.64898/2026.04.28.721095; this version posted April 30, 2026. The copyright holder for this preprint (which was not certified by peer review) is the author/funder, who has granted bioRxiv a license to display the preprint in perpetuity. It is made available under aCC-BY 4.0 International license.

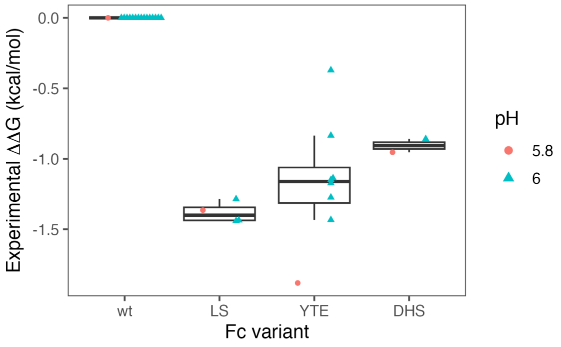


Supplemental Figure 6. Experimental $\Delta\Delta G$ values relative to *wt* for pH 5.8 Fc variant binding affinity measurements from Lee, et al. [14] compared to pH 6.0 $\Delta\Delta G$ values for the same variants from other sources.

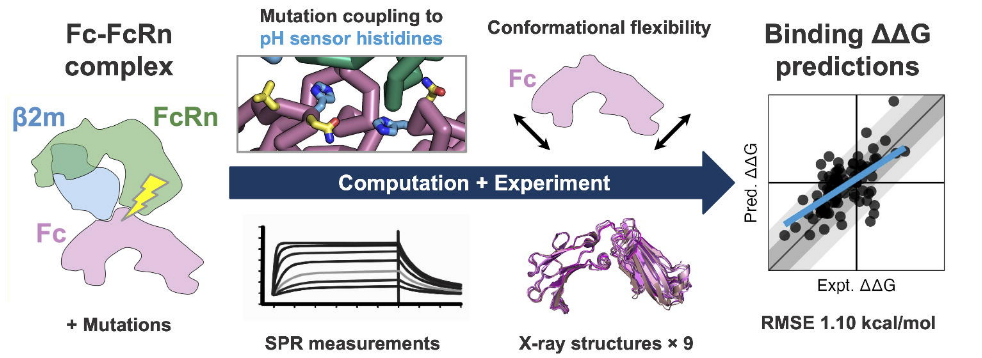
Fc-FcRn complex
β2m
FcRn
Fc
+ Mutations
Mutation coupling to
pH sensor histidines
Conformational flexibility
Fc
Computation + Experiment
SPR measurements
X-ray structures × 9
Binding ΔΔG predictions
Pred. ΔΔG
Expt. ΔΔG
RMSE 1.10 kcal/mol
